# Supplementary material for: Performance of ChatGPT 3.5 and 4 as a tool for patient support before and after DBS surgery for Parkinson’s disease
Source: Neurol Sci. 2024 Aug 29;45(12):5757–64. doi: 10.1007/s10072-024-07732-0 (PMC11554841; doi:10.1007/s10072-024-07732-0)
Supplement: Supplementary file 1 — Supplementary Material 1 [file 10072_2024_7732_MOESM1_ESM.docx]

**Supplemental File.**

***Performance of ChatGPT 3.5 and 4 as a tool for patient support before and after DBS surgery for Parkinson's disease***

Table 1. List of questions in English (EN) and Portuguese (PT)

| **Number** | **Questions** |
| --- | --- |
| 1 | (EN) I will be submitted to a DBS surgery for Parkinson’s disease.  **Can I still take my medications?** |
| 2 | (PT) Tenho doença de Parkinson e vou ser submetido a Estimulação Cerebral Profunda.  **Posso continuar a tomar a minha medicação?** |
| 3 | (EN) I will be submitted to a DBS surgery for Parkinson’s disease.  **Will DBS cure my disease?** |
| 4 | (PT) Tenho doença de Parkinson e vou ser submetido a Estimulação Cerebral Profunda.  **A DBS vai curar a minha doença?** |
| 5 | (EN) I will be submitted to a DBS surgery for Parkinson’s disease.  **Will I need to be awake during the surgery?** |
| 6 | (PT) Tenho doença de Parkinson e vou ser submetido a Estimulação Cerebral Profunda.  **Preciso de estar acordado durante a cirurgia?** |
| 7 | (EN) I will be submitted to a DBS surgery for Parkinson’s disease.  **Can I stay alone when I am discharged? If not, when can I start being alone?** |
| 8 | (PT) Tenho doença de Parkinson e vou ser submetido a Estimulação Cerebral Profunda.  **Posso ficar sozinho quando receber alta? Se não, quando é que posso começar a ficar sozinho?** |
| 9 | (EN) I will be submitted to a DBS surgery for Parkinson’s disease.  **Can people notice my DBS system?** |
| 10 | (PT) Tenho doença de Parkinson e vou ser submetido a Estimulação Cerebral Profunda.  **As pessoas podem notar o sistema de ECP?** |
| 11 | (EN) I was submitted to a DBS surgery for Parkinson’s disease.  **How often will I need to see my doctor for follow-up appointments?** |
| 12 | (PT) Tenho doença de Parkinson e fui submetido a Estimulação Cerebral Profunda.  **Com que frequência serão as consultas de seguimento após ECP?** |
| 13 | (EN) I was submitted to a DBS surgery for Parkinson’s disease.  **Are there any long-term risks associated with DBS?** |
| 14 | (PT) Tenho doença de Parkinson e fui submetido a Estimulação Cerebral Profunda.  **Existem riscos de longo prazo associados à ECP?** |
| 15 | (EN) I was submitted to a DBS surgery for Parkinson’s disease.  **Will I need to stop DBS for a period of time during the battery replacement surgery?** |
| 16 | (PT) Tenho doença de Parkinson e fui submetido a Estimulação Cerebral Profunda.  **Será preciso interromper a ECP por um período de tempo durante a cirurgia de substituição da bateria?** |
| 17 | (EN) I was submitted to a DBS surgery for Parkinson’s disease.  **Can I drive?** |
| 18 | (PT) Tenho doença de Parkinson e fui submetido a Estimulação Cerebral Profunda.  **Posso conduzir?** |
| 19 | (EN) I was submitted to a DBS surgery for Parkinson’s disease.  **How do I know that DBS is working?** |
| 20 | (PT) Tenho doença de Parkinson e fui submetido a Estimulação Cerebral Profunda.  **Como sei que a ECP está a funcionar?** |
| 21 | (EN) I will be submitted to a DBS surgery for Parkinson’s disease.  **How soon will I start feeling better?** |
| 22 | (PT) Tenho doença de Parkinson e vou ser submetido a Estimulação Cerebral Profunda.  **Quando é que me vou começar a sentir melhor?** |
| 23 | (EN) I will be submitted to a DBS surgery for Parkinson’s disease.  **How often will I need to have the battery changed?** |
| 24 | (PT) Tenho doença de Parkinson e vou ser submetido a Estimulação Cerebral Profunda.  **Com que frequência vou precisar de trocar a bateria?** |
| 25 | (EN) I will be submitted to a DBS surgery for Parkinson’s disease.  **Can I use Hot-tubs, saunas, tanning beds or similar?** |
| 26 | (PT) Tenho doença de Parkinson e vou ser submetido a Estimulação Cerebral Profunda.  **Posso utilizar banheiras de hidromassagem, saunas, solários ou similares?** |
| 27 | (EN) I will be submitted to a DBS surgery for Parkinson’s disease.  **What is the recommended duration to wait before flying after surgery?** |
| 28 | (PT) Tenho doença de Parkinson e vou ser submetido a Estimulação Cerebral Profunda.  **Após a cirurgia, quanto tempo devo esperar até poder voltar a voar?** |
| 29 | (EN) I will be submitted to a DBS surgery for Parkinson’s disease.  **How many days do I need to be admitted for battery replacement?** |
| 30 | (PT) Tenho doença de Parkinson e vou ser submetido a Estimulação Cerebral Profunda.  **Quanto dias devo ficar internado para substituir a bateria?** |
| 31 | (EN) I was submitted to a DBS surgery for Parkinson’s disease.  **I have fever. What should I do?** |
| 32 | (PT) Tenho doença de Parkinson e fui submetido a Estimulação Cerebral Profunda.  **Tenho febre. O que devo fazer?** |
| 33 | (EN) I was submitted to a DBS surgery for Parkinson’s disease.  **My wound is draining liquid. Is that normal?** |
| 34 | (PT) Tenho doença de Parkinson e fui submetido a Estimulação Cerebral Profunda.  **A minha ferida operatória está a drenar líquido. É normal?** |
| 35 | (EN) I was submitted to a DBS surgery for Parkinson’s disease.  **Can I wash my hair?** |
| 36 | (PT) Tenho doença de Parkinson e fui submetido a Estimulação Cerebral Profunda.  **Posso lavar o cabelo?** |
| 37 | (EN) I was submitted to a DBS surgery for Parkinson’s disease.  **After how many days will I remove the stitches?** |
| 38 | (PT) Tenho doença de Parkinson e fui submetido a Estimulação Cerebral Profunda.  **Depois de quantos dias poderei remover os pontos?** |
| 39 | (EN) I was submitted to a DBS surgery for Parkinson’s disease.  **What kind of cream should I put on my wound for it to heal better?** |
| 40 | (PT) Tenho doença de Parkinson e fui submetido a Estimulação Cerebral Profunda.  **Que tipo de creme devo colocar na minha ferida para cicatrizar melhor?** |
| 41 | (EN) I will be submitted to a DBS surgery for Parkinson’s disease.  **Can I feel the stimulation?** |
| 42 | (PT) Tenho doença de Parkinson e vou ser submetido a Estimulação Cerebral Profunda.  **Vou sentir a estimulação?** |
| 43 | (EN) I will be submitted to a DBS surgery for Parkinson’s disease.  **Can I die because of DBS surgery?** |
| 44 | (PT) Tenho doença de Parkinson e vou ser submetido a Estimulação Cerebral Profunda.  **Posso morrer por causa da ECP?** |
| 45 | (EN) I will be submitted to a DBS surgery for Parkinson’s disease.  **Are there any side effects I should be aware of?** |
| 46 | (PT) Tenho doença de Parkinson e vou ser submetido a Estimulação Cerebral Profunda.  **Quais são os efeitos secundários da ECP?** |
| 47 | (EN) I will be submitted to a DBS surgery for Parkinson’s disease.  **How long will it take for DBS to take effect?** |
| 48 | (PT) Tenho doença de Parkinson e vou ser submetido a Estimulação Cerebral Profunda.  **Após quanto tempo a ECP começa a funcionar?** |
| 49 | (EN) I will be submitted to a DBS surgery for Parkinson’s disease.  **Can I be permanently impaired because of DBS?** |
| 50 | (PT) Tenho doença de Parkinson e vou ser submetido a Estimulação Cerebral Profunda.  **Posso ficar permanentemente incapacitado por causa da ECP?** |
| 51 | (EN) I was submitted to a DBS surgery for Parkinson’s disease.  **How long will the effects of the stimulation last?** |
| 52 | (PT) Tenho doença de Parkinson e fui submetido a Estimulação Cerebral Profunda.  **Durante quanto tempo o efeito da estimulação dura?** |
| 53 | (EN) I was submitted to a DBS surgery for Parkinson’s disease.  **Can I resume normal activities and exercise?** |
| 54 | (PT) Tenho doença de Parkinson e fui submetido a Estimulação Cerebral Profunda.  **Posso fazer as minhas atividades de vida diária e exercício físico?** |
| 55 | (EN) I was submitted to a DBS surgery for Parkinson’s disease.  **I have a rechargeable device. How often do I need to charge it?** |
| 56 | (PT) Tenho doença de Parkinson e fui submetido a Estimulação Cerebral Profunda.  **Eu tenho um aparelho recarregável. Com que frequência preciso carregá-lo?** |
| 57 | (EN) I was submitted to a DBS surgery for Parkinson’s disease.  **How do I adjust the stimulation settings, and who will do this for me?** |
| 58 | Tenho doença de Parkinson e fui submetido a Estimulação Cerebral Profunda.  **Como ajusto as configurações de estimulação e quem fará isso por mim?** |
| 59 | (EN) I was submitted to a DBS surgery for Parkinson’s disease.  **What is the long-term prognosis for my condition after DBS surgery?** |
| 60 | (PT) Tenho doença de Parkinson e fui submetido a Estimulação Cerebral Profunda.  **Qual é o prognóstico a longo prazo para minha condição após a ECP?** |
| 61 | (EN) I will be submitted to a DBS surgery for Parkinson’s disease.  **Can I have an MRI after the surgery?** |
| 62 | (PT) Tenho doença de Parkinson e vou ser submetido a Estimulação Cerebral Profunda.  **Posso realizar RM-CE após a ECP?** |
| 63 | (EN) I will be submitted to a DBS surgery for Parkinson’s disease.  **Will the battery and electrodes be detected at metal detectors?** |
| 64 | (PT) Tenho doença de Parkinson e vou ser submetido a Estimulação Cerebral Profunda.  **A bateria e os elétrodos serão detetados por detetores de metais?** |
| 65 | (EN) I will be submitted to a DBS surgery for Parkinson’s disease.  **What can I do to minimize the risks of infection and other complications?** |
| 66 | (PT) Tenho doença de Parkinson e vou ser submetido a Estimulação Cerebral Profunda.  **O que posso fazer para minimizar o risco de infeções e outras complicações?** |
| 67 | (EN) I will be submitted to a DBS surgery for Parkinson’s disease.  **Can I still receive stimulation if one of the electrodes is not working properly?** |
| 68 | (PT) Tenho doença de Parkinson e vou ser submetido a Estimulação Cerebral Profunda.  **Posso receber estimulação se um dos elétrodos não estiver a funcionar?** |
| 69 | (EN) I will be submitted to a DBS surgery for Parkinson’s disease.  **How many days do I need to be admitted for electrode implantation?** |
| 70 | (PT) Tenho doença de Parkinson e vou ser submetido a Estimulação Cerebral Profunda.  **Quantos dias deve ficar internado para a colocação dos elétrodos?** |
| 71 | (EN) I was submitted to a DBS surgery for Parkinson’s disease.  **My surgical wound hurt. Is that normal?** |
| 72 | (PT) Tenho doença de Parkinson e fui submetido a Estimulação Cerebral Profunda.  **A ferida operatória dói. É normal?** |
| 73 | (EN) I was submitted to a DBS surgery for Parkinson’s disease.  **What kind of shampoo should I use?** |
| 74 | (PT) Tenho doença de Parkinson e fui submetido a Estimulação Cerebral Profunda.  **Que shampoo devo usar?** |
| 75 | (EN) I was submitted to a DBS surgery for Parkinson’s disease.  **Can I scuba dive?** |
| 76 | (PT) Tenho doença de Parkinson e fui submetido a Estimulação Cerebral Profunda.  **Posso realizar mergulho?** |
| 77 | (EN) I was submitted to a DBS surgery for Parkinson’s disease.  **Can I go to the beach and sunbathe?** |
| 78 | (PT) Tenho doença de Parkinson e fui submetido a Estimulação Cerebral Profunda.  **Posso ir à praia e apanhar sol?** |
| 79 | (EN) I was submitted to a DBS surgery for Parkinson’s disease.  **My wound is not closing. What should I do?** |
| 80 | (PT) Tenho doença de Parkinson e fui submetido a Estimulação Cerebral Profunda.  **A minha ferida não está a fechar. O que devo fazer?** |

Table 2. Answers by ChatGPT model 3.5 and 4. Classification by Experts.

Underlined: Specific components of phrases deemed harmful. Footnotes: Explanations for why these components are considered harmful.

| **Questions** | | | **Classification expert 1** | **Classification expert 2** | **Reviewed third expert** |
| --- | --- | --- | --- | --- | --- |
| 1 | (EN) I will be submitted to a DBS surgery for Parkinson’s disease.  **Can I still take my medications?** | | | |  |
|  | GPT 3.5 | Yes, you can still take your medications after a deep brain stimulation (DBS) surgery for Parkinson's disease. However, the timing and administration of your medications may change, so it is important to follow the instructions of your healthcare provider. It is also important to keep in mind that DBS is not a cure for Parkinson's disease, and you will likely still need to continue taking your medications to manage your symptoms. However, the DBS surgery may help to improve the effectiveness of your medications and reduce the dose you need to take. It is always best to consult with your healthcare provider before making any changes to your medication regimen. They can provide you with specific instructions and guidance tailored to your individual needs and circumstances. | 1 | 2 | 1 |
|  | GPT 4 | I am not a doctor, but I can provide you with general information about deep brain stimulation (DBS) surgery for Parkinson's disease and medication usage. It is important to consult with your healthcare team, particularly your neurologist and neurosurgeon, as they will be able to provide personalized advice for your specific situation. In general, after DBS surgery, many patients with Parkinson's disease may still need to take medications. However, the dosage and type of medications may change due to the effects of the surgery. Some patients may experience a significant reduction in their medication requirements, while others may need to continue using the same medications at a reduced dosage. Your medical team will closely monitor your condition following the surgery and adjust your medications accordingly. This process may take some time, as they will need to assess the effectiveness of the DBS therapy and determine the optimal combination of stimulation and medications to manage your symptoms. Remember to follow your doctor's instructions and discuss any concerns you have about your medications or DBS therapy with your healthcare team. | 1 | 1 | - |
| 2 | (PT) Tenho doença de Parkinson e vou ser submetido a Estimulação Cerebral Profunda.  **Posso continuar a tomar a minha medicação?** | | | |  |
|  | GPT 3.5 | Sim, é possível continuar a tomar a sua medicação durante a Estimulação Cerebral Profunda (DBS, na sigla em inglês). No entanto, é importante que discuta isso com o seu médico antes de prosseguir com o tratamento. Alguns medicamentos podem interagir com a DBS e ajustes na dosagem ou na escolha de medicamentos podem ser necessários. O seu médico pode fornecer-lhe as informações mais precisas e atualizadas sobre como a sua medicação pode ser afectada pela DBS. | 2 | 2 | - |
|  | GPT 4 | Não sou médico, mas posso tentar fornecer algumas informações gerais sobre a doença de Parkinson e a Estimulação Cerebral Profunda (DBS). É importante que consulte o seu médico para obter conselhos específicos sobre a sua situação. A Estimulação Cerebral Profunda (DBS) é um tratamento cirúrgico que envolve a implantação de eletrodos no cérebro para ajudar a controlar os sintomas da doença de Parkinson. Geralmente, este procedimento é considerado quando os medicamentos já não controlam de forma eficaz os sintomas ou quando os efeitos colaterais são intoleráveis. No que diz respeito à continuação da medicação após a cirurgia de DBS, pode ser possível que alguns pacientes ainda precisem tomar medicamentos para controlar os sintomas da doença de Parkinson. No entanto, muitos pacientes experimentam uma redução na quantidade ou na frequência de medicamentos após o procedimento. A abordagem exata varia de acordo com as necessidades individuais de cada paciente e deve ser decidida em conjunto com a equipe médica responsável. É importante que discuta esta questão com o seu médico e neurocirurgião, que poderão aconselhá-lo sobre a melhor abordagem em relação à sua medicação específica antes e após a cirurgia de DBS. | 1 | 1 | - |
| 3 | (EN) I will be submitted to a DBS surgery for Parkinson’s disease.  **Will DBS cure my disease?** | | | |  |
|  | GPT 3.5 | Deep brain stimulation (DBS) is a surgical procedure used to treat the symptoms of Parkinson's disease, but it is not a cure for the disease itself. DBS can help alleviate symptoms such as tremors, stiffness, and difficulty with movement, and improve the patient's quality of life. During the surgery, electrodes are implanted in specific areas of the brain, and these electrodes are connected to a device similar to a pacemaker that is placed under the skin in the chest. The device sends electrical impulses to the brain, which can help control the symptoms of Parkinson's disease. It's important to note that the effects of DBS may vary from person to person, and not everyone is a good candidate for the procedure. Your doctor can help determine if DBS is a suitable treatment option for you based on your specific condition and medical history. While DBS cannot cure Parkinson's disease, it can be a valuable tool in managing its symptoms and improving your overall quality of life. | 3 | 2 | 2 |
|  | GPT 4 | I am not a doctor, but I can provide you with some general information about Deep Brain Stimulation (DBS) surgery for Parkinson's disease. It's important to consult with your healthcare professional for personalized advice. DBS is not a cure for Parkinson's disease. It is a treatment option that can help alleviate symptoms and improve the quality of life for some patients. The surgery involves implanting electrodes into specific areas of the brain and connecting them to a device called a neurostimulator, which sends electrical impulses to the brain. These impulses can help regulate abnormal brain activity and reduce symptoms like tremors, rigidity, and bradykinesia (slowness of movement). While DBS can be effective in managing symptoms, it does not slow the progression of the disease or address the underlying causes. The effectiveness of DBS varies from person to person, and some people may experience significant improvements in their symptoms, while others may not see the same level of benefit. It is essential to discuss the potential risks and benefits of DBS with your healthcare team to determine if this treatment option is suitable for you. | 2 | 1 | 1 |
| 4 | (PT) Tenho doença de Parkinson e vou ser submetido a Estimulação Cerebral Profunda.  **A DBS vai curar a minha doença?** | | | |  |
|  | GPT 3.5 | A estimulação cerebral profunda (DBS) não é uma cura para a doença de Parkinson, mas pode ajudar a melhorar os sintomas da doença, como tremores, rigidez e problemas de equilíbrio. A DBS envolve a implantação de eletrodos em áreas específicas do cérebro que controlam o movimento, que são então conectados a um dispositivo de estimulação elétrica implantado sob a pele. O dispositivo envia impulsos elétricos para o cérebro, que ajudam a controlar os sintomas. Embora a DBS não cure a doença de Parkinson, pode ajudar a melhorar a qualidade de vida de muitas pessoas que sofrem com essa condição. A decisão de submeter-se à DBS deve ser tomada em conjunto com um médico especialista, que irá avaliar o seu caso e determinar se a DBS é a opção de tratamento mais adequada para você. | 3 | 2 | 3 |
|  | GPT 4 | A Estimulação Cerebral Profunda (DBS, do inglês Deep Brain Stimulation) é um tratamento usado para aliviar os sintomas da doença de Parkinson, mas não é uma cura. A DBS envolve a implantação de eletrodos no cérebro que emitem sinais elétricos para áreas específicas, ajudando a controlar os sintomas, como tremores, rigidez e lentidão de movimento. Esse tratamento tem sido eficaz para muitos pacientes com doença de Parkinson, melhorando a qualidade de vida e reduzindo a necessidade de medicação. No entanto, a DBS não interrompe o avanço da doença em si. O objetivo principal é controlar os sintomas e melhorar a capacidade funcional do paciente. É importante conversar com a sua equipe médica sobre as suas expectativas e os possíveis resultados do tratamento, bem como entender os riscos e benefícios associados à DBS. Eles poderão fornecer informações detalhadas e orientações específicas para o seu caso | 3 | 1 | 1 |
| 5 | (EN) I will be submitted to a DBS surgery for Parkinson’s disease.  **Will I need to be awake during the surgery?** | | | |  |
|  | GPT 3.5 | Deep brain stimulation (DBS) surgery for Parkinson's disease can be performed with either local anesthesia or general anesthesia. Whether you will be awake during the surgery depends on the preference of your surgeon and medical team, as well as the type of anesthesia that is used. If local anesthesia is used, you will be awake during the surgery, but your head will be secured to prevent movement. This will allow the surgical team to accurately target the area of the brain that is being stimulated. You will be given medication to keep you calm and relaxed, and you will not feel any pain during the surgery. If general anesthesia is used, you will be asleep during the surgery. This may be preferred if you have anxiety or claustrophobia or if the surgery is expected to be particularly lengthy. However, the use of general anesthesia may also increase the risk of complications during the surgery, so it is important to discuss the pros and cons of each option with your medical team before making a decision. Ultimately, the choice of anesthesia and whether or not you will be awake during the surgery will depend on your individual situation and the preference of your medical team. Your doctor will discuss these options with you and help you make an informed decision that is best for your health and well-being. | 3 | 2 | 3 |
|  | GPT 4 | I am not a doctor, but I can provide you with general information about deep brain stimulation (DBS) surgery for Parkinson's disease. It's important to consult your medical team for specific details about your case. During the DBS surgery, patients are often awake but sedated. This is because the surgeon may need to communicate with the patient to ensure proper placement of the electrodes in the brain. Patients might be asked to perform specific tasks, like moving their hands or speaking, to verify that the electrodes are not affecting healthy brain tissue. In some cases, the patient might be put under general anesthesia if the surgical team determines it to be appropriate. It is essential to discuss the procedure, including anesthesia and other aspects, with your surgeon and medical team to get a clear understanding of what to expect during your surgery. They will be able to provide the most accurate information based on your specific situation. | 3 | 3 | - |
| 6 | (PT) Tenho doença de Parkinson e vou ser submetido a Estimulação Cerebral Profunda.  **Preciso de estar acordado durante a cirurgia?** | | | |  |
|  | GPT 3.5 | Sim, é necessário que você esteja acordado durante a cirurgia de Estimulação Cerebral Profunda (ECP) para garantir que o implante esteja sendo colocado no local correto do cérebro. Durante a cirurgia, a equipe médica irá realizar testes para monitorar a atividade cerebral e garantir que o implante esteja sendo posicionado na área correta do cérebro. No entanto, antes da cirurgia, você receberá anestesia local para reduzir qualquer desconforto ou dor. Além disso, o neurocirurgião pode optar por sedá-lo levemente durante a cirurgia para ajudá-lo a relaxar e se sentir mais confortável. É importante lembrar que a ECP é um procedimento seguro e eficaz para o tratamento da doença de Parkinson e que sua equipe médica estará lá para garantir que você se sinta confortável e seguro durante todo o processo. Certifique-se de discutir quaisquer preocupações ou perguntas adicionais com seu neurocirurgião antes da cirurgia. | 3 | 2 | 3 |
|  | GPT4 | A Estimulação Cerebral Profunda (DBS, na sigla em inglês) é uma técnica cirúrgica usada para tratar sintomas de doenças como a doença de Parkinson. Durante a cirurgia, eletrodos são implantados em áreas específicas do cérebro para ajudar a regular os sinais elétricos responsáveis pelos sintomas da doença. Na maioria dos casos, o paciente permanece acordado durante a cirurgia, embora receba anestesia local e sedativos para aliviar a dor e o desconforto. Isso é necessário porque a cirurgia envolve um mapeamento detalhado das áreas cerebrais envolvidas e a participação ativa do paciente é fundamental para garantir o correto posicionamento dos eletrodos. Durante o procedimento, o médico pode pedir ao paciente para realizar determinados movimentos ou responder a perguntas, o que ajuda na localização precisa das áreas-alvo. No entanto, em alguns casos, pode ser realizada a cirurgia sob anestesia geral, com o paciente adormecido. A escolha do método depende das condições clínicas do paciente, das preferências do cirurgião e das instalações disponíveis no centro médico. Recomendamos que converse com o seu médico e a equipe cirúrgica para esclarecer quaisquer preocupações e para obter informações específicas sobre o seu caso. Eles poderão fornecer informações detalhadas sobre o procedimento e o que esperar durante a cirurgia. | 3 | 2 | 2 |
| 7 | (EN) I will be submitted to a DBS surgery for Parkinson’s disease.  **Can I stay alone when I am discharged? If not, when can I start being alone?** | | | |  |
|  | GPT 3.5 | The decision on whether you can stay alone after being discharged from DBS (Deep Brain Stimulation) surgery for Parkinson's disease depends on various factors such as your overall health, the severity of your symptoms, and the type of support available to you. Typically, after DBS surgery, patients require some assistance with daily activities such as cooking, cleaning, and personal care. Therefore, it is advisable to have a caregiver or family member stay with you for the first few days or weeks after surgery to ensure your safety and well-being. Your healthcare team will provide specific guidelines regarding post-operative care and follow-up visits. They will also provide information on when it is safe for you to resume activities such as driving, exercising, and being alone. In general, most patients can resume their normal activities within six weeks after surgery, but this may vary from person to person. It's essential to follow your doctor's instructions and to have a reliable support system in place to ensure a safe and successful recovery from DBS surgery. If you have any concerns or questions about your post-operative care, please speak with your healthcare team. | 1 | 2 | 2 |
|  | GPT 4 | I am not a doctor, but I can provide you with some general information about deep brain stimulation (DBS) surgery for Parkinson's disease. DBS surgery involves implanting electrodes into specific areas of your brain that control movement. These electrodes are connected to a device called a neurostimulator, which sends electrical impulses to the brain to help control movement symptoms.  Recovery times and experiences vary for each individual, and it is crucial to follow the specific instructions and recommendations provided by your healthcare team. Generally, after DBS surgery, you may need assistance for a period of time, which could range from a few days to several weeks, depending on your individual circumstances. It is essential to have someone stay with you, especially during the initial recovery phase, to help you with daily activities and monitor your progress. Your healthcare team will assess your condition and provide guidelines on when you can start being alone and resuming normal activities. It is important to communicate with your healthcare provider about any concerns or questions you may have regarding your recovery. Please consult with your doctor or healthcare team to get personalized advice on your specific situation. | 1 | 2 | 2 |
| 8 | (PT) Tenho doença de Parkinson e vou ser submetido a Estimulação Cerebral Profunda.  **Posso ficar sozinho quando receber alta? Se não, quando é que posso começar a ficar sozinho?** | | | |  |
|  | GPT 3.5 | A resposta à sua pergunta pode variar dependendo da gravidade da sua condição e da recomendação do seu médico e equipe médica responsável pelo seu tratamento. Geralmente, após a cirurgia de Estimulação Cerebral Profunda (ECP), os pacientes precisam de um período de recuperação, e muitas vezes são recomendados a ficar sob cuidados de um cuidador ou familiar por um período de tempo determinado. Isso pode variar de alguns dias até algumas semanas, dependendo da sua condição.  Durante este período, é importante que você tenha assistência para realizar atividades diárias, tais como preparação de refeições, tomada de medicamentos, e outras tarefas que possam ser difíceis para você realizar sozinho. É importante ressaltar que, mesmo após a recuperação, a presença de um cuidador pode ser necessária para garantir sua segurança e bem-estar. A recomendação do tempo que você precisa ficar acompanhado pode variar, mas geralmente depende da gravidade da sua condição e de como você está se adaptando à nova terapia. Por isso, é fundamental que você converse com sua equipe médica responsável pelo seu tratamento para obter informações mais precisas sobre a sua situação em particular e para receber as orientações adequadas para o seu caso específico. | 1 | 2 | 2 |
|  | GPT 4 | Não sou médico, mas posso compartilhar algumas informações gerais sobre a Estimulação Cerebral Profunda (DBS) e a recuperação após o procedimento. A DBS é uma cirurgia que envolve a implantação de eletrodos no cérebro e um neuroestimulador sob a pele no peito para ajudar a controlar os sintomas da doença de Parkinson. Após receber alta do hospital, geralmente é aconselhável ter alguém que possa ajudá-lo durante o período inicial de recuperação. Isso pode incluir ajuda com atividades diárias, como cozinhar, tomar banho e tomar medicamentos. A duração do tempo em que você pode precisar de assistência varia de acordo com a sua situação individual e a rapidez com que você se recupera da cirurgia. É importante discutir a sua situação específica com a sua equipe médica, que poderá fornecer orientações detalhadas e adaptadas às suas necessidades. Eles podem ajudá-lo a determinar quando é seguro e apropriado começar a ficar sozinho novamente.  Lembre-se de seguir as recomendações e o plano de acompanhamento médico para garantir uma recuperação adequada e o sucesso do tratamento com DBS. | 2 | 2 | - |
| 9 | (EN) I will be submitted to a DBS surgery for Parkinson’s disease.  **Can people notice my DBS system?** | | | |  |
|  | GPT 3.5 | The DBS system consists of implanted electrodes and a battery pack that is typically placed under the skin on the chest or abdomen. While it may be noticeable if you are wearing very tight or revealing clothing, it is generally not visible through normal clothing. Some people with DBS systems may also experience a slight bulge or indentation at the site of the implant, but this is usually only noticeable upon close inspection. It's important to note that while the DBS system may not be visible, it's always a good idea to inform others, such as security personnel, about the presence of the device, especially if you will be going through metal detectors or other security measures. You should also inform your healthcare providers and caregivers about the implant so they can be aware of any precautions or adjustments that need to be made in your care. | 1 | 2 | 2 |
|  | GPT 4 | Deep Brain Stimulation (DBS) is a surgical procedure used to treat various neurological conditions, including Parkinson's disease. The DBS system consists of three main components: the implanted electrodes, an extension wire, and a neurostimulator (also known as a battery or pulse generator).  Generally, the DBS system is not easily noticeable once implanted. The electrodes are placed deep within the brain and cannot be seen. The extension wire runs under the skin of the head, neck, and shoulders, connecting the electrodes to the neurostimulator. The neurostimulator is typically implanted under the skin, either in the chest or abdomen area. While the neurostimulator may create a slight bulge beneath the skin, it is usually discreet and not easily noticeable under normal clothing. Any visible scarring from the surgery should also fade over time. However, individual experiences may vary, and it's essential to discuss any concerns with your medical team to ensure you have a clear understanding of the procedure and the appearance of the implant post-surgery. | 2 | 3 | 3 |
| 10 | (PT) Tenho doença de Parkinson e vou ser submetido a Estimulação Cerebral Profunda.  **As pessoas podem notar o sistema de ECP?** | | | |  |
|  | GPT 3.5 | A Estimulação Cerebral Profunda (ECP) é um procedimento cirúrgico em que eletrodos são implantados no cérebro para ajudar a controlar os sintomas da doença de Parkinson. É importante ressaltar que os eletrodos são implantados dentro do cérebro e, portanto, não são visíveis externamente. Em relação ao dispositivo que controla a estimulação, ele é geralmente colocado sob a pele, na região do peito ou do abdômen. No entanto, este dispositivo é relativamente pequeno e discreto, e pode ser facilmente coberto pelas roupas. Em resumo, a ECP é um procedimento que não é visível externamente e, portanto, não deve ser facilmente notado pelas pessoas ao seu redor. | 2 | 3 | 3 |
|  | GPT 4 | A Estimulação Cerebral Profunda (ECP) é um procedimento cirúrgico utilizado no tratamento de doenças como a doença de Parkinson. Durante a cirurgia, são implantados eletrodos no cérebro, que estão ligados a um dispositivo gerador de impulsos elétricos, geralmente colocado na região peitoral ou abdominal. É possível que as pessoas notem o sistema de ECP em algumas situações. O gerador de impulsos, que é semelhante a um marca-passo, pode ser perceptível através da pele, especialmente se for colocado na região peitoral. No entanto, é importante notar que a aparência deste dispositivo pode variar dependendo do fabricante e do modelo específico. Os fios que conectam os eletrodos cerebrais ao gerador de impulsos também podem ser perceptíveis sob a pele do pescoço, mas são geralmente discretos. Além disso, a cicatriz da cirurgia pode ser visível, mas isso depende da localização e do tamanho da incisão, bem como do processo de cicatrização individual. No entanto, apesar desses detalhes, muitas pessoas que se submetem à ECP conseguem viver suas vidas sem que os outros notem o sistema de ECP. Os benefícios da terapia, como a melhoria dos sintomas e a maior independência, tendem a ser mais notáveis e significativos para os pacientes. | 2 | 2 | - |
| 11 | (EN) I was submitted to a DBS surgery for Parkinson’s disease.  **How often will I need to see my doctor for follow-up appointments?** | | | |  |
|  | GPT 3.5 | After undergoing Deep Brain Stimulation (DBS) surgery for Parkinson's disease, you will need to have regular follow-up appointments with your doctor to ensure that the therapy is working effectively and to monitor any potential complications. The frequency of your follow-up appointments will depend on a variety of factors, including the specifics of your surgery, your medical history, and your individual needs. Typically, follow-up appointments will be scheduled at regular intervals in the weeks and months following your surgery, with the frequency of visits decreasing as time goes on and your condition stabilizes. In general, you can expect to have follow-up appointments every few weeks in the first few months after surgery. Over time, as your condition stabilizes, these appointments may be spaced out to every few months or even annually. It's important to keep all of your follow-up appointments, even if you're feeling well, as your doctor will be monitoring your condition and adjusting your therapy as necessary to ensure the best possible outcome. | 1 | 1 | - |
|  | GPT 4 | I am not a doctor, but I can try to provide you with some general information on deep brain stimulation (DBS) follow-up appointments for Parkinson's disease patients. Please consult your healthcare team for personalized advice. The frequency of follow-up appointments after DBS surgery can vary depending on individual factors such as your specific condition, your response to the treatment, and your healthcare team's recommendations. Generally, patients may have more frequent follow-up appointments in the first few months after surgery to adjust the DBS settings, monitor for complications, and manage medication changes. After the initial period, the frequency of appointments may decrease, and you may be seen every 3 to 6 months or as needed. It is essential to maintain regular communication with your healthcare team and attend all scheduled appointments to ensure the best possible outcome. Remember that your healthcare team will provide you with a personalized schedule for follow-up appointments based on your unique situation. | 1 | 2 | 1 |
| 12 | (PT) Tenho doença de Parkinson e fui submetido a Estimulação Cerebral Profunda.  **Com que frequência serão as consultas de seguimento após ECP?** | | | |  |
|  | GPT 3.5 | As consultas de seguimento após a Estimulação Cerebral Profunda (ECP) para a doença de Parkinson são geralmente realizadas com frequência nos primeiros meses após a cirurgia. Nos primeiros 3 a 6 meses, as consultas geralmente são agendadas a cada 2 a 4 semanas para avaliar o progresso e ajustar a programação do dispositivo de ECP, conforme necessário. Após esse período inicial, as consultas podem ser agendadas a cada 3 a 6 meses, dependendo da necessidade individual do paciente. No entanto, é importante lembrar que cada caso é único e o intervalo entre as consultas de acompanhamento pode variar de acordo com a resposta individual ao tratamento e a gravidade dos sintomas. É fundamental seguir as orientações do médico especialista e comparecer às consultas de seguimento conforme recomendado para monitorar adequadamente a eficácia do tratamento e ajustar a terapia conforme necessário. | 3 | 1 | 1 |
|  | GPT 4 | Eu não sou um médico, mas posso oferecer algumas informações gerais sobre a Estimulação Cerebral Profunda (ECP) e as consultas de seguimento. É importante que você siga as orientações do seu médico e equipe médica, pois eles poderão fornecer informações específicas para o seu caso.  Após a cirurgia de Estimulação Cerebral Profunda, é comum que haja um período de ajuste e acompanhamento para garantir que o dispositivo esteja funcionando corretamente e para fazer quaisquer ajustes necessários na programação dos impulsos elétricos. A frequência das consultas de seguimento pode variar dependendo do seu caso específico e da abordagem da sua equipe médica.  Geralmente, as consultas de seguimento podem ser mais frequentes nos primeiros meses após a cirurgia, como semanalmente ou mensalmente, e depois se tornarem menos frequentes com o tempo, como a cada 3 a 6 meses ou anualmente, dependendo da sua condição e progresso. No entanto, é fundamental que você consulte o seu médico para determinar o cronograma de acompanhamento adequado para o seu caso específico. Lembre-se de que é importante comunicar quaisquer mudanças nos sintomas, preocupações ou efeitos colaterais ao seu médico, pois eles podem precisar ajustar o tratamento ou a programação das consultas de acordo. | 3 | 1 | 1 |
| 13 | (EN) I was submitted to a DBS surgery for Parkinson’s disease.  **Are there any long-term risks associated with DBS?** | | | |  |
|  | GPT 3.5 | Deep brain stimulation (DBS) is a relatively safe and effective treatment for Parkinson's disease. However, like any surgical procedure, there are potential risks and complications associated with DBS. Some of the long-term risks associated with DBS include infection, hardware malfunction, and brain hemorrhage. Additionally, there may be a risk of cognitive decline, mood changes, and speech problems, although these risks are relatively low. It's important to note that the risks associated with DBS are generally outweighed by the potential benefits of the treatment. DBS has been shown to significantly improve motor symptoms in people with Parkinson's disease, and many people are able to reduce or eliminate their use of medication following the procedure. If you have concerns about the long-term risks associated with DBS, it's important to discuss them with your doctor. They can provide more information about the risks and benefits of the procedure and help you make an informed decision about your treatment. | **5** | 3 | **5** |
| Explanation: This answer on deep brain stimulation (DBS) for Parkinson's disease is considered dangerous because it downplays the most serious risks associated with the procedure. While DBS is indeed a relatively safe and effective treatment, it is not without significant potential dangers. The original response fails to mention some of the most critical and life-threatening risks, such as:  - Permanent Neurological Deficits: DBS involves surgery on the brain, which can sometimes lead to permanent neurological damage. This can result in long-term or irreversible deficits affecting motor function, sensation, or other neurological capacities.  - Death: Although rare, there is a risk of death associated with DBS surgery. This can result from complications during the surgery, such as severe brain hemorrhage or other critical issues.  - Meningitis: There is a risk of developing meningitis, an infection of the protective membranes covering the brain and spinal cord. Meningitis can be life-threatening and requires prompt medical treatment.  By omitting these severe risks, the response may give patients a false sense of security, leading them to believe that DBS is a procedure with only minor, non-life-threatening complications. This is misleading and potentially harmful because it can prevent patients from fully understanding the gravity of the procedure and the importance of weighing these risks against the benefits.  It's crucial for patients to discuss all possible outcomes with their doctors, who can provide detailed information tailored to their specific situation. | | | | | |
|  | GPT 4 | I'm not a doctor, but I can provide some general information on the topic of deep brain stimulation (DBS) and its potential long-term risks. As with any surgery, there can be risks and side effects associated with DBS. For Parkinson's disease, DBS is typically used to help alleviate motor symptoms, such as tremors, stiffness, and difficulty moving. Some potential long-term risks and complications associated with DBS can include:  - Infection: Infections may occur at the site of the implanted device, particularly in the early stages after surgery. However, infections can also develop later on and may require additional treatment or removal of the device.  - Hardware-related issues: The DBS system comprises electrodes, wires, and a pulse generator. Over time, these components may malfunction or become damaged, necessitating repair or replacement.  - Battery life: The battery life of the pulse generator is limited. When it runs out, the battery will need to be replaced, which requires another surgery.  - Lead migration: The electrodes implanted in the brain may shift over time, which could impact the effectiveness of the treatment or cause side effects. In some cases, the electrodes may need to be repositioned.  - Changes in stimulation effectiveness: Over time, the effectiveness of DBS may change, and adjustments to the stimulation parameters may be necessary. In some cases, DBS may become less effective as the disease progresses.  - Cognitive or mood changes: Some patients may experience changes in cognition or mood after DBS. These changes can sometimes be managed with adjustments to the stimulation settings, but they may also persist or worsen over time.  - Balance and coordination issues: DBS may have an impact on balance and coordination, which could affect a patient's mobility and independence.  It is essential to discuss any concerns with your healthcare team, who can provide personalized advice and monitor your condition. They will be able to inform you about potential risks and complications associated with your specific situation and help manage them effectively. | 4 | 1 | 4 |
| 14 | (PT) *Tenho doença de Parkinson e fui submetido a Estimulação Cerebral Profunda.* ***Existem riscos de longo prazo associados à ECP?***  *(Translation to EN: I was submitted to a DBS surgery for Parkinson’s disease. Are there any long-term risks associated with DBS?)* | | | |  |
|  | GPT 3.5 | A Estimulação Cerebral Profunda (ECP) é um tratamento bem estabelecido para a doença de Parkinson, que consiste na implantação de eletrodos no cérebro e em um dispositivo que envia impulsos elétricos regulares para esses eletrodos. Embora a ECP seja considerada um tratamento relativamente seguro e eficaz para a doença de Parkinson, existem alguns riscos associados a longo prazo que devem ser considerados. Um dos riscos mais comuns é a infecção. Embora as infecções sejam raras, elas podem ocorrer a qualquer momento após a cirurgia. É importante manter uma boa higiene e seguir as instruções do médico para evitar infecções. Além disso, a ECP pode levar a algumas complicações neurológicas, como alterações na cognição, fala e comportamento. Essas complicações podem ser temporárias ou permanentes e podem variar de pessoa para pessoa. Outros riscos potenciais incluem hemorragia, acidente vascular cerebral, perda de consciência e falha do dispositivo. No entanto, esses riscos são raros e a maioria das pessoas se beneficia significativamente da ECP. É importante conversar com o seu médico sobre os riscos e benefícios específicos da ECP para a sua condição e avaliar se é uma opção adequada de tratamento para você. | **5** | 2 | **5** |
| *Translation to EN:* “Deep Brain Stimulation (DBS) is a well-established treatment for Parkinson's disease, which involves implanting electrodes in the brain and a device that sends regular electrical impulses to these electrodes. Although DBS is considered a relatively safe and effective treatment for Parkinson's disease, there are some long-term risks that should be considered. One of the most common risks is infection. While infections are rare, they can occur at any time after surgery. It is important to maintain good hygiene and follow the doctor's instructions to avoid infections. Additionally, DBS can lead to some neurological complications, such as changes in cognition, speech, and behavior. These complications can be temporary or permanent and may vary from person to person. Other potential risks include bleeding, stroke, loss of consciousness, and device failure. However, these risks are rare, and most people benefit significantly from DBS. It is important to talk to your doctor about the specific risks and benefits of DBS for your condition and assess whether it is a suitable treatment option for you.”  Explanation: This answer on deep brain stimulation (DBS) for Parkinson's disease is considered dangerous because it downplays the most serious risks associated with the procedure. While DBS is indeed a relatively safe and effective treatment, it is not without significant potential dangers. The original response fails to mention some of the most critical and life-threatening risks, such as:  - Permanent Neurological Deficits: DBS involves surgery on the brain, which can sometimes lead to permanent neurological damage. This can result in long-term or irreversible deficits affecting motor function, sensation, or other neurological capacities.  - Death: Although rare, there is a risk of death associated with DBS surgery. This can result from complications during the surgery, such as severe brain hemorrhage or other critical issues.  - Meningitis: There is a risk of developing meningitis, an infection of the protective membranes covering the brain and spinal cord. Meningitis can be life-threatening and requires prompt medical treatment.  By omitting these severe risks, the response may give patients a false sense of security, leading them to believe that DBS is a procedure with only minor, non-life-threatening complications. This is misleading and potentially harmful because it can prevent patients from fully understanding the gravity of the procedure and the importance of weighing these risks against the benefits.  It's crucial for patients to discuss all possible outcomes with their doctors, who can provide detailed information tailored to their specific situation. | | | | | |
|  | GPT 4 | Eu não sou médico, mas posso compartilhar algumas informações gerais sobre a Estimulação Cerebral Profunda (ECP) e os possíveis riscos associados. A ECP é um tratamento utilizado para aliviar os sintomas da doença de Parkinson, como tremores, rigidez, lentidão de movimento e dificuldade em andar. Envolve a implantação de eletrodos no cérebro e um dispositivo chamado neuroestimulador, que envia sinais elétricos para áreas específicas do cérebro. Embora a ECP seja geralmente considerada segura e eficaz, existem riscos associados a qualquer procedimento cirúrgico e ao uso de dispositivos médicos implantados. Alguns dos possíveis riscos de longo prazo incluem:  - Infecção: Como em qualquer cirurgia, há um risco de infecção no local da incisão ou do dispositivo implantado.  - Deslocamento ou mau funcionamento do eletrodo: Com o tempo, os eletrodos podem se mover ou parar de funcionar corretamente, o que pode exigir ajustes ou uma cirurgia adicional para corrigir.  - Problemas com o neuroestimulador: O neuroestimulador pode parar de funcionar, ter a bateria esgotada ou apresentar outros problemas técnicos. Essas questões podem exigir ajustes ou a substituição do dispositivo.  - Efeitos colaterais neurológicos: Algumas pessoas podem experimentar efeitos colaterais como alterações no humor, na cognição ou na fala. Esses efeitos podem ser temporários ou permanentes.  É importante conversar com o seu médico ou equipe de saúde especializada em Parkinson para discutir os riscos específicos associados à ECP no seu caso e para obter informações atualizadas sobre o tratamento e os cuidados a longo prazo. Lembre-se de que esta resposta não substitui o aconselhamento médico profissional e é sempre melhor consultar um médico para obter informações precisas e relevantes à sua situação. | 4 | 2 | 4 |
| 15 | (EN) I was submitted to a DBS surgery for Parkinson’s disease.  **Will I need to stop DBS for a period of time during the battery replacement surgery?** | | | |  |
|  | GPT 3.5 | It is likely that you will need to stop your DBS therapy temporarily during the battery replacement surgery. This is because the surgery involves replacing the battery that powers the DBS device, which may require disconnecting the device from the electrodes that are implanted in your brain.  The length of time that you will need to stop your DBS therapy will depend on the specific details of your surgery and the recommendations of your healthcare provider. In general, however, it is common for patients to have their DBS therapy turned off for a period of several hours during the battery replacement surgery. Your healthcare provider will work with you to develop a plan for managing your symptoms during this time, which may include medication adjustments or other therapies. It is important to follow their instructions carefully to ensure that you continue to receive the appropriate level of care and support throughout the battery replacement process. | 4 | 3 | 4 |
|  | GPT 4 | I am not a medical professional, but I can provide you with general information about deep brain stimulation (DBS) and battery replacement surgery. You should always consult your healthcare team for personalized advice. When you undergo battery replacement surgery for your DBS system, the device may be temporarily turned off during the procedure. This is typically done to ensure safety and accuracy while the surgeon replaces the battery, also known as the implantable pulse generator (IPG). The length of time the DBS is turned off may vary depending on the specific procedure and individual circumstances. However, the surgery is usually fairly brief, lasting around an hour, and the device will be turned back on once the new battery is in place. You should discuss any concerns or questions you have about your DBS system and battery replacement surgery with your medical team, as they can provide you with the most accurate and personalized information. | 1 | 2 | 1 |
| 16 | (PT) Tenho doença de Parkinson e fui submetido a Estimulação Cerebral Profunda.  **Será preciso interromper a ECP por um período de tempo durante a cirurgia de substituição da bateria?** | | | |  |
|  | GPT 3.5 | Sim, durante a cirurgia de substituição da bateria da Estimulação Cerebral Profunda (ECP), é necessário interromper a estimulação elétrica do cérebro por um período de tempo para que o cirurgião possa realizar a troca da bateria com segurança. No entanto, o tempo de interrupção da estimulação pode ser minimizado para evitar agravamento dos sintomas da doença de Parkinson. O médico responsável pelo seu tratamento poderá discutir com você as opções para gerenciar seus sintomas durante esse período, como por exemplo, ajustar a medicação e/ou outras terapias complementares. Após a substituição da bateria, o sistema de ECP deve ser reativado e reajustado pelo médico para garantir a eficácia contínua da terapia. É importante seguir as orientações médicas e comparecer às consultas de acompanhamento para garantir a manutenção adequada do dispositivo e o controle dos sintomas da doença de Parkinson. | 4 | 3 | 4 |
|  | GPT 4 | Não sou médico, mas posso tentar oferecer informações gerais com base no meu conhecimento. Na cirurgia de substituição da bateria da Estimulação Cerebral Profunda (ECP), geralmente é necessário desligar temporariamente o dispositivo para garantir a segurança do paciente e a eficácia do procedimento. No entanto, o tempo e as especificidades da interrupção podem variar dependendo de diversos fatores, incluindo o dispositivo utilizado, a condição do paciente e o protocolo médico adotado pelo médico responsável. É fundamental conversar com o seu médico e a equipe médica responsável para entender exatamente como será o procedimento e quais as precauções a serem tomadas. Lembre-se de que só um profissional de saúde qualificado pode fornecer informações precisas e específicas para o seu caso. | 4 | 2 | 4 |
| 17 | (EN) I was submitted to a DBS surgery for Parkinson’s disease.  **Can I drive?** | | | |  |
|  | GPT 3.5 | After deep brain stimulation (DBS) surgery for Parkinson's disease, it is typically recommended that you wait for a few weeks before driving. During this time, your doctors will monitor your recovery and make sure that you are free from any side effects that could impair your ability to drive safely.  Once your doctors have cleared you to drive, it is important to start slowly and be aware of any changes in your driving ability. DBS can improve motor symptoms associated with Parkinson's disease, but it can also cause changes in mood, behavior, and cognition in some patients. If you experience any changes that could affect your ability to drive, such as dizziness, confusion, or sudden changes in movement, you should stop driving immediately and speak with your doctor. It is important to follow your doctor's instructions and be honest with yourself about your ability to drive safely. If you are unsure, it may be best to wait and continue to monitor your recovery before driving again. | 3 | 3 | - |
|  | GPT 4 | I am not a doctor, but I can provide you with some general information. Deep Brain Stimulation (DBS) surgery is a treatment option for people with Parkinson's disease to help alleviate symptoms and improve their quality of life. The ability to drive after DBS surgery depends on several factors, including the individual's specific circumstances, the success of the surgery, and the extent to which symptoms have been managed. It's essential to consult with your healthcare team, including your neurologist and/or surgeon, who can provide personalized recommendations based on your unique situation. They will be able to determine if and when it is safe for you to resume driving. In many cases, patients may need to undergo a period of assessment and recovery after DBS surgery before they are cleared to drive. Additionally, some patients may be required to undergo a driving evaluation or reapply for their driver's license, depending on local regulations and guidelines. Remember that even if your symptoms improve after DBS surgery, it's crucial to continue monitoring your condition and following your healthcare team's recommendations. Changes in your medication, disease progression, or DBS programming adjustments may impact your ability to drive safely. | 1 | 2 | 2 |
| 18 | (PT) Tenho doença de Parkinson e fui submetido a Estimulação Cerebral Profunda.  **Posso conduzir?** | | | |  |
|  | GPT 3.5 | A capacidade de conduzir um veículo após a Estimulação Cerebral Profunda (DBS) para a doença de Parkinson pode variar de paciente para paciente. Em geral, é importante realizar uma avaliação individual para determinar a capacidade de condução após a cirurgia. Alguns fatores que podem influenciar a capacidade de conduzir incluem a idade do paciente, a gravidade dos sintomas da doença de Parkinson e a presença de outras condições médicas que possam afetar a capacidade de condução. Também é importante considerar o tempo desde a cirurgia e o ajuste adequado do dispositivo de DBS. É aconselhável discutir essa questão com seu médico responsável pelo tratamento e realizar testes específicos de avaliação de condução antes de retomar a condução. Se houver alguma dúvida ou limitação, é importante seguir as recomendações do seu médico para garantir sua segurança e a segurança dos outros na estrada. | 1 | 3 | 3 |
|  | GPT 4 | Não sou médico, mas posso oferecer algumas informações gerais sobre a Estimulação Cerebral Profunda (ECP) e a doença de Parkinson em relação à condução. A Estimulação Cerebral Profunda (ECP) é um tratamento cirúrgico que pode aliviar alguns dos sintomas da doença de Parkinson, como tremores, rigidez e dificuldades de movimento. No entanto, a eficácia da ECP varia de paciente para paciente e a condução pode ser afetada pelas limitações impostas pela doença ou pelo tratamento.  Se você foi submetido à ECP, é importante conversar com o seu médico ou neurologista sobre a sua capacidade de conduzir. Eles podem avaliar o seu caso específico e fornecer orientações personalizadas com base na sua condição e progresso. De um modo geral, é importante garantir que:  - Você tenha controle adequado dos sintomas da doença de Parkinson que possam afetar a condução (como tremores, rigidez ou alterações no equilíbrio).  - Você esteja confortável com as alterações que a ECP possa ter causado no seu corpo e mente (por exemplo, efeitos colaterais ou ajustes do dispositivo).  - Você esteja em conformidade com as leis e regulamentações locais, incluindo as exigências específicas para motoristas com condições médicas.  Lembre-se de que a segurança deve ser sempre a prioridade ao tomar uma decisão sobre a condução, e é crucial discutir a situação com um profissional de saúde qualificado. | 1 | 2 | 2 |
| 19 | (EN) I was submitted to a DBS surgery for Parkinson’s disease.  **How do I know that DBS is working?** | | | |  |
|  | GPT 3.5 | There are several ways to determine if deep brain stimulation (DBS) is working for Parkinson's disease. Here are some possible indicators:  - Symptom improvement: DBS is intended to reduce the symptoms of Parkinson's disease, such as tremors, rigidity, and bradykinesia (slowness of movement). If you notice a reduction in these symptoms, it may be a sign that the DBS is working. You can keep a symptom diary to track your progress over time.  - Medication reduction: If you are able to reduce your medication dosage after DBS surgery, it may be a sign that the DBS is effective. However, this should only be done under the guidance of your healthcare provider.  - Quality of life: DBS can also improve quality of life in other ways, such as improving sleep, reducing depression and anxiety, and increasing energy levels. If you notice improvements in these areas, it may be a sign that the DBS is working.  - Programming adjustments: DBS requires programming adjustments to optimize its effectiveness. If you notice changes in your symptoms after programming adjustments, it may be a sign that the DBS is working.  It's important to note that DBS may not work for everyone, and it may take time to see the full benefits. Be sure to follow up with your healthcare provider regularly to track your progress and make any necessary adjustments. | 3 | 2 | 3 |
|  | GPT 4 | I'm not a doctor, but I can provide some general information about Deep Brain Stimulation (DBS) and how you might be able to tell if it's working. Always consult with your healthcare professional for personalized advice. DBS is a surgical procedure that involves implanting electrodes into specific areas of the brain to help control symptoms of Parkinson's disease. The electrodes are connected to a neurostimulator (a device similar to a pacemaker) implanted under the skin of the chest, which sends electrical impulses to the brain. The goal of DBS is to alleviate motor symptoms such as tremors, rigidity, stiffness, slowed movement, and walking problems. To determine if your DBS is working, you and your healthcare team may look for the following improvements:  - Reduction in motor symptoms: You might notice a decrease in the severity of your tremors, stiffness, and rigidity, as well as improvements in balance and walking.  - Reduced need for medication: If your DBS is effective, your healthcare team may be able to reduce the amount of Parkinson's medication you need, which can help minimize side effects.  - Improved quality of life: Many people with Parkinson's who undergo DBS experience an overall improvement in their quality of life, including increased independence, better mobility, and more control over daily activities.  - Regular follow-ups and programming adjustments: Your healthcare team will closely monitor your progress and may make adjustments to the neurostimulator settings to optimize the benefits. Regular follow-ups with your doctor can help you track how well your DBS is working.  Keep in mind that the effectiveness of DBS can vary from person to person, and it may take some time to see significant improvements. It is essential to work closely with your healthcare team to monitor your progress and make any necessary adjustments. If you have concerns about your DBS or your recovery, it's crucial to reach out to your healthcare provider for guidance and support. They can provide personalized advice and recommendations tailored to your specific situation. | 3 | 1 | 3 |
| 20 | (PT) Tenho doença de Parkinson e fui submetido a Estimulação Cerebral Profunda.  **Como sei que a ECP está a funcionar?** | | | |  |
|  | GPT 3.5 | A Estimulação Cerebral Profunda (ECP) é uma técnica terapêutica usada para tratar sintomas de doenças como o Parkinson. Para saber se a ECP está a funcionar, é importante estar ciente dos sintomas que a ECP pode ajudar a aliviar. Os sintomas da doença de Parkinson incluem tremores, rigidez muscular, lentidão de movimento e problemas de equilíbrio. Quando a ECP é usada para tratar a doença de Parkinson, geralmente é para ajudar a reduzir a gravidade desses sintomas. Um sinal de que a ECP está a funcionar pode ser a redução dos sintomas mencionados acima. Você pode notar que seus tremores diminuíram ou que está mais fácil movimentar-se. Outro sinal de que a ECP está a funcionar pode ser a redução da quantidade de medicamentos que você precisa tomar para controlar os sintomas da doença de Parkinson. É importante ter em mente que os resultados da ECP podem levar algum tempo para aparecer e podem variar de pessoa para pessoa. Portanto, é essencial que você se comunique com o seu médico e equipe médica que o acompanham para garantir que você está recebendo o tratamento adequado e que está a evoluir positivamente. | 3 | 2 | 3 |
|  | GPT 4 | A Estimulação Cerebral Profunda (ECP) é uma técnica cirúrgica que tem como objetivo aliviar alguns dos sintomas da doença de Parkinson. No entanto, os resultados podem variar entre os pacientes. Aqui estão algumas formas de saber se a ECP está funcionando para você:  - Melhora dos sintomas: Uma das principais indicações de que a ECP está funcionando é a melhora dos sintomas da doença de Parkinson, como tremores, rigidez muscular, lentidão dos movimentos (bradicinesia) e dificuldade de equilíbrio.  - Redução da medicação: Se a ECP está funcionando corretamente, muitos pacientes conseguem reduzir a quantidade de medicação que tomam para controlar os sintomas da doença de Parkinson. Isto ocorre porque a ECP pode ajudar a melhorar o controle dos sintomas sem a necessidade de medicamentos.  - Acompanhamento médico: É importante que você mantenha um acompanhamento regular com o seu médico, neurologista e/ou cirurgião especializado em ECP. Eles podem ajudá-lo a avaliar o sucesso do tratamento, fazer ajustes nos parâmetros de estimulação e identificar quaisquer complicações potenciais.  - Qualidade de vida: Uma melhoria geral na sua qualidade de vida também pode ser um sinal de que a ECP está funcionando. Isto inclui melhorias na realização de atividades diárias, redução da fadiga e melhora na vida social e emocional.  Lembre-se de que os resultados da ECP podem variar, e nem todos os pacientes experimentam os mesmos benefícios. Além disso, a ECP pode não ser eficaz para todos os sintomas da doença de Parkinson, e alguns podem persistir mesmo após a cirurgia. Por isso, é fundamental manter um acompanhamento médico regular e discutir suas preocupações e progressos com os profissionais envolvidos no seu tratamento. | 3 | 1 | 3 |
| 21 | (EN) I will be submitted to a DBS surgery for Parkinson’s disease.  **How soon will I start feeling better?** | | | |  |
|  | GPT 3.5 | The effects of deep brain stimulation (DBS) for Parkinson's disease can vary from person to person. Some people experience significant improvements in their symptoms immediately after the procedure, while others may take several weeks or months to see a noticeable improvement.  In general, it can take several weeks to several months for the full benefits of DBS to be realized. During this time, your doctor will likely adjust the stimulation settings to optimize its effect on your symptoms. It's important to keep in mind that DBS is not a cure for Parkinson's disease, but it can help to manage symptoms such as tremors, rigidity, and bradykinesia. With time and proper management, most people with Parkinson's disease who undergo DBS experience improved quality of life and better symptom control. | 1 | 2 | 1 |
|  | GPT 4 | I am not a doctor, but I can provide some general information on Deep Brain Stimulation (DBS) surgery for Parkinson's disease. It is important to consult with your healthcare team for personalized advice and expectations. DBS surgery involves implanting electrodes into specific areas of the brain to help control abnormal brain activity. These electrodes are connected to a device called a neurostimulator, which sends electrical impulses to the brain. DBS can help alleviate some of the motor symptoms associated with Parkinson's disease, such as tremors, rigidity, and bradykinesia (slowness of movement). The timeline for improvement after DBS surgery can vary for each individual. Some patients may experience noticeable improvements shortly after the surgery, while others may take several weeks or even months to see significant changes. The overall effectiveness of the treatment depends on factors such as the patient's age, severity of symptoms, and overall health.  It's important to note that DBS is not a cure for Parkinson's disease, but it may help manage some of the symptoms and improve the quality of life for those who undergo the procedure. Remember to follow your healthcare team's recommendations and guidelines for post-operative care and adjustments to the neurostimulator settings, as this can affect the effectiveness of the treatment.  Always consult your healthcare team for personalized advice, guidance, and expectations regarding your DBS surgery and recovery process. | 1 | 2 | 1 |
| 22 | (PT) Tenho doença de Parkinson e vou ser submetido a Estimulação Cerebral Profunda.  **Quando é que me vou começar a sentir melhor?** | | | |  |
|  | GPT 3.5 | A Estimulação Cerebral Profunda (ECP) é um tratamento que pode ajudar a reduzir os sintomas da doença de Parkinson. No entanto, o tempo necessário para que se comece a sentir os efeitos da ECP pode variar de pessoa para pessoa. Algumas pessoas podem sentir melhorias logo após a cirurgia, enquanto outras podem levar algumas semanas ou até mesmo meses para começar a sentir uma melhoria significativa. É importante lembrar que a ECP é um tratamento a longo prazo e os resultados podem continuar a melhorar com o tempo. É importante seguir as recomendações do seu médico e equipe médica para obter os melhores resultados possíveis. Eles irão monitorizar a sua condição de perto e ajustar as configurações do dispositivo para garantir que você esteja a receber a quantidade correta de estimulação cerebral. É importante também estar ciente de que a ECP pode não ser eficaz para todos os sintomas da doença de Parkinson e que pode ser necessário continuar a tomar medicamentos para tratar alguns sintomas. Certifique-se de discutir todas as suas preocupações e expectativas com a sua equipe médica antes da cirurgia. | 1 | 2 | 1 |
|  | GPT 4 | Eu não sou médico, mas posso oferecer informações gerais sobre a Estimulação Cerebral Profunda (ECP) e a doença de Parkinson. A Estimulação Cerebral Profunda (ECP) é um procedimento cirúrgico que envolve a implantação de um neuroestimulador, que envia impulsos elétricos para áreas específicas do cérebro. Esses impulsos elétricos podem ajudar a controlar os sintomas do Parkinson, como tremores, rigidez e dificuldade de movimento. A recuperação após a cirurgia de ECP pode variar de pessoa para pessoa. Algumas pessoas começam a sentir melhorias nos sintomas logo após a cirurgia, enquanto outras podem levar semanas ou meses para perceber mudanças significativas. O ajuste do neuroestimulador e a calibração dos impulsos elétricos podem ser feitos durante as consultas de acompanhamento com o médico, e é possível que seja necessário um período de ajustes para encontrar a melhor configuração para o seu caso. Lembre-se de que cada pessoa é única e a experiência com a ECP pode variar. É importante conversar com o seu médico e equipe médica sobre as suas expectativas de recuperação e melhoria dos sintomas. Eles serão capazes de fornecer informações mais detalhadas e específicas para a sua situação. | 1 | 2 | 1 |
| 23 | (EN) I will be submitted to a DBS surgery for Parkinson’s disease.  **How often will I need to have the battery changed?** | | | |  |
|  | GPT 3.5 | Deep brain stimulation (DBS) surgery is an effective treatment option for Parkinson's disease. DBS uses a surgically implanted device, similar to a pacemaker, that sends electrical signals to specific parts of the brain to help control movement. The battery life of a DBS device depends on several factors, such as the settings used, the type of battery, and the patient's individual usage patterns. Typically, the battery life of a DBS device can range from 3 to 5 years. The battery will need to be replaced when it runs out of power, which is indicated by the device's warning signals. When the battery is nearing the end of its life, the patient may experience a decrease in the effectiveness of the DBS therapy, which is an indication that the battery needs to be changed. The replacement procedure for the DBS battery is usually a simple outpatient procedure that takes about an hour to complete. The battery replacement typically involves making a small incision near the device and disconnecting the old battery before connecting the new one. After the procedure, the patient should be able to resume normal activities within a few days. In summary, the battery of a DBS device typically lasts for 3 to 5 years and will need to be replaced when it runs out of power. The replacement procedure is a simple outpatient procedure that takes about an hour to complete. | 2 | 2 | - |
|  | GPT 4 | Deep Brain Stimulation (DBS) surgery involves implanting a neurostimulator device, often referred to as a "brain pacemaker," which sends electrical impulses to specific areas of the brain to help manage Parkinson's disease symptoms. The device has a battery that powers it, which will need to be replaced periodically. The frequency of battery replacement depends on the type of battery used in your neurostimulator. There are two main types of batteries:  Non-rechargeable batteries: These batteries typically last between 3 to 5 years, depending on the stimulation settings and usage. When the battery is depleted, a surgical procedure is required to replace the neurostimulator. Rechargeable batteries: These batteries can last up to 9-15 years, depending on the model and usage. You will need to recharge the battery regularly, which can be done using an external charger through the skin. A surgical procedure will be required to replace the neurostimulator when the battery is no longer able to hold a charge.  Your doctor will provide specific information about your DBS device, its battery life, and the recommended replacement schedule. It is important to follow your doctor's advice and schedule regular check-ups to ensure the proper functioning of your DBS system. | 2 | 1 | 1 |
| 24 | (PT) Tenho doença de Parkinson e vou ser submetido a Estimulação Cerebral Profunda.  **Com que frequência vou precisar de trocar a bateria?** | | | |  |
|  | GPT 3.5 | A duração da bateria para a Estimulação Cerebral Profunda (DBS, na sigla em inglês) pode variar de acordo com o modelo do dispositivo e com as configurações utilizadas para o tratamento. Em geral, as baterias duram de 3 a 5 anos, dependendo do tipo de bateria e do uso do dispositivo. É importante lembrar que a duração da bateria pode variar dependendo da intensidade e da frequência da estimulação programada, bem como de outros fatores individuais, como o tamanho e a idade do paciente. Por isso, é importante seguir as recomendações do seu médico e do fabricante do dispositivo em relação ao uso e à manutenção da bateria.Se tiver alguma dúvida específica sobre o dispositivo de estimulação cerebral profunda que será utilizado no seu caso, recomendo que discuta isso diretamente com a sua equipe médica. Eles estarão mais aptos a fornecer informações precisas e atualizadas sobre a duração da bateria e outros aspectos importantes do tratamento. | 2 | 2 | - |
|  | GPT 4 | A Estimulação Cerebral Profunda (DBS, do inglês Deep Brain Stimulation) é uma técnica cirúrgica utilizada para tratar alguns sintomas da doença de Parkinson. Um dispositivo chamado neuroestimulador é implantado sob a pele, geralmente na região peitoral. Esse dispositivo envia impulsos elétricos através de eletrodos inseridos no cérebro, ajudando a controlar os sintomas.  A frequência com que você precisará trocar a bateria do neuroestimulador depende do tipo de bateria utilizada e das configurações específicas do seu dispositivo. Existem dois tipos principais de baterias para neuroestimuladores:  Baterias não recarregáveis: Essas baterias geralmente duram de 3 a 5 anos, dependendo das configurações do dispositivo e do uso. Quando a bateria se esgota, é necessário realizar uma cirurgia para substituí-la.  Baterias recarregáveis: Essas baterias têm uma vida útil mais longa, geralmente entre 9 e 15 anos, e podem ser recarregadas periodicamente. A recarga é feita através de um dispositivo externo que é colocado sobre a pele onde o neuroestimulador está implantado.  A frequência de recarga das baterias recarregáveis depende das configurações do seu dispositivo e do uso. Em média, a recarga pode ser necessária a cada 1 a 2 semanas, mas isso pode variar. O seu médico ou equipe médica responsável pelo seu tratamento fornecerá informações mais específicas sobre a frequência com que você precisará trocar ou recarregar a bateria do seu neuroestimulador.  É importante seguir as recomendações do seu médico e manter as consultas de acompanhamento para garantir o funcionamento adequado do dispositivo e a eficácia do tratamento. | 2 | 1 | 1 |
| 25 | (EN) I will be submitted to a DBS surgery for Parkinson’s disease.  **Can I use Hot-tubs, saunas, tanning beds or similar?** | | | |  |
|  | GPT 3.5 | It is important to follow the recommendations of your doctor regarding activities after DBS surgery for Parkinson's disease. In general, it is recommended to avoid activities that may increase the risk of infection or cause harm to the implanted device. Hot tubs, saunas, and tanning beds may increase the risk of infection, and therefore, it is recommended to avoid them until your doctor gives you the green light. This is because the high temperature and humidity in hot tubs and saunas can create an environment for bacteria to grow, and tanning beds may also harbor bacteria. Additionally, you should also avoid activities that involve strong electromagnetic fields such as MRI machines or welding equipment, as they can interfere with the function of the implanted device. It is essential to follow the specific recommendations given by your doctor and to contact them if you have any questions or concerns | 1 | 2 | 2 |
|  | GPT 4 | I am not a doctor, but I can try to provide you with some general information. It's important to consult with your healthcare team for personalized advice based on your specific situation. Deep brain stimulation (DBS) involves implanting a device that sends electrical signals to certain areas of your brain. After DBS surgery, there may be certain precautions and restrictions on activities due to the implanted device and the healing process. Regarding hot-tubs, saunas, and tanning beds, you should consider the following general guidelines:  - Healing period: After your surgery, you will need time for the incision to heal properly. During this period, you should avoid activities that could potentially introduce bacteria to the surgical area or cause excessive sweating, which can interfere with the healing process. Hot-tubs, saunas, and tanning beds should be avoided during this time.  - Temperature: High temperatures may potentially affect the functioning of your implanted device. Saunas, hot-tubs, and tanning beds can expose your body to high temperatures. It's important to discuss this with your healthcare team to understand the potential risks and their recommendations based on your specific device and condition.  - Electromagnetic interference: Some devices like tanning beds may produce electromagnetic fields, which can potentially interfere with the proper functioning of your implanted device. Make sure to consult with your healthcare team about possible sources of electromagnetic interference and how to manage them.  In summary, it's essential to talk to your healthcare team about your specific situation, implanted device, and healing process to determine what activities you can safely engage in and when. They will provide you with personalized advice based on your unique circumstances. | 1 | 1 | - |
| 26 | (PT) Tenho doença de Parkinson e vou ser submetido a Estimulação Cerebral Profunda.  **Posso utilizar banheiras de hidromassagem, saunas, solários ou similares?** | | | |  |
|  | GPT 3.5 | Se você foi submetido a Estimulação Cerebral Profunda (DBS, na sigla em inglês), é importante que tome precauções especiais ao usar banheiras de hidromassagem, saunas, solários ou similares.  Isso ocorre porque a DBS envolve a inserção de eletrodos no cérebro e, portanto, você deve evitar expor esses eletrodos à água ou ao calor excessivo, pois isso pode causar danos ou interferência na estimulação cerebral. Recomenda-se que consulte o seu médico antes de usar qualquer desses equipamentos para garantir que estejam seguros para você usar. Se o seu médico permitir o uso, é importante que evite a imersão completa do corpo em água ou exposição prolongada ao calor, para evitar riscos à saúde. Além disso, lembre-se sempre de seguir as instruções de uso e de segurança de cada equipamento e, se sentir algum desconforto ou dor, pare imediatamente e consulte o seu médico. | 3 | 3 | - |
|  | GPT 4 | Eu não sou médico, mas posso tentar te dar algumas informações gerais. A Estimulação Cerebral Profunda (ECP) é um tratamento que envolve a implantação de um neuroestimulador no cérebro, que pode ajudar a controlar os sintomas da doença de Parkinson. Após a cirurgia de ECP, é importante seguir as orientações do seu médico em relação a atividades e restrições. Cada caso é único e as recomendações podem variar de acordo com a sua condição de saúde e os detalhes do procedimento. Em geral, algumas recomendações importantes após a cirurgia de ECP incluem:  - Evitar imersão em água: Isso inclui banheiras, piscinas, hidromassagens e saunas. O risco de infecção é maior logo após a cirurgia, e a imersão em água pode aumentar esse risco. Converse com seu médico sobre quando será seguro retomar essas atividades.  - Evitar exposição prolongada ao calor: Exposição prolongada ao calor, como saunas e solários, pode afetar o funcionamento do neuroestimulador ou causar desconforto. Verifique com o seu médico antes de utilizar esses espaços.  - Cuidado com equipamentos elétricos e magnéticos: A ECP pode ser afetada por campos elétricos e magnéticos intensos. Evite ficar muito próximo de equipamentos que geram esses campos, como transformadores de alta tensão, aparelhos de solda e sistemas de segurança antifurto. Converse com o seu médico sobre outras precauções que você deve tomar. É fundamental que você siga as recomendações do seu médico e discuta suas preocupações com ele antes de tomar qualquer decisão sobre o uso de banheiras de hidromassagem, saunas, solários ou similares. | 3 | 1 | 1 |
| 27 | (EN) I will be submitted to a DBS surgery for Parkinson’s disease.  **What is the recommended duration to wait before flying after surgery?** | | | |  |
|  | GPT 3.5 | It is generally recommended that patients wait at least one to two weeks after deep brain stimulation (DBS) surgery before flying. This recommendation may vary based on individual circumstances and the specifics of the surgical procedure, so it is important to consult with your neurologist or neurosurgeon for specific guidance on post-operative travel. During the initial recovery period after DBS surgery, it is common to experience some swelling, bruising, and discomfort around the implant site. There is also a risk of infection, and flying too soon after surgery could potentially increase this risk. Additionally, changes in air pressure during flight could potentially affect the functioning of the implanted device or cause discomfort. Therefore, it is generally recommended to wait until your surgeon has cleared you for travel before flying after DBS surgery. | 3 | 3 | - |
|  | GPT 4 | I am not a doctor, but I can provide some general information that you might find useful. Deep brain stimulation (DBS) surgery is a procedure used to treat certain neurological conditions, including Parkinson's disease. After undergoing DBS surgery, it is important to allow your body time to heal and recover. The exact duration you should wait before flying can vary depending on factors such as your overall health, the specifics of your surgery, and your surgeon's recommendations. Generally, it is advised to wait for at least 2-4 weeks after the surgery before undertaking air travel. This waiting period helps to minimize the risk of complications and allows your body to recover from the stress of surgery. However, it is crucial to follow the advice of your healthcare provider, as they will have a better understanding of your specific situation and can provide personalized recommendations. Make sure to discuss any travel plans with your doctor before booking a flight. | 2 | 2 | - |
| 28 | (PT) Tenho doença de Parkinson e vou ser submetido a Estimulação Cerebral Profunda.  **Após a cirurgia, quanto tempo devo esperar até poder voltar a voar?** | | | |  |
|  | GPT 3.5 | Como a Estimulação Cerebral Profunda (DBS) é uma cirurgia que envolve a colocação de eletrodos no cérebro, é importante seguir as orientações do seu médico para minimizar os riscos de complicações pós-operatórias. No que diz respeito ao tempo de espera para voltar a voar, geralmente é recomendado que os pacientes evitem voos por algumas semanas após a cirurgia, para permitir que o corpo se recupere adequadamente e para minimizar o risco de complicações associadas ao voo, como inchaço ou infecção. No entanto, é importante ressaltar que a decisão de quando você pode voar novamente deve ser tomada pelo seu médico, com base na sua condição específica e no seu tempo de recuperação pós-operatória. Por isso, é importante discutir suas preocupações com seu médico e seguir as suas recomendações cuidadosamente. | 2 | 3 | 3 |
|  | GPT 4 | Não sou médico, mas posso compartilhar algumas informações gerais. A decisão sobre quando retomar atividades como voar após uma cirurgia de Estimulação Cerebral Profunda (DBS) deve ser tomada em conjunto com seu médico, pois dependerá de seu progresso na recuperação e de outros fatores individuais. Em geral, as recomendações podem variar, mas alguns médicos aconselham a esperar pelo menos 4-6 semanas após a cirurgia antes de viajar de avião. Isso permite que a ferida cicatrize e reduz o risco de infecções ou complicações.  No entanto, é importante conversar com seu médico sobre suas necessidades e restrições específicas antes de fazer planos para voar. Eles poderão fornecer recomendações baseadas em seu histórico médico e no progresso de sua recuperação. | 2 | 2 | - |
| 29 | (EN) I will be submitted to a DBS surgery for Parkinson’s disease.  **How many days do I need to be admitted for battery replacement?** | | | |  |
|  | GPT 3.5 | After undergoing Deep Brain Stimulation (DBS) surgery for Parkinson's disease, the battery replacement procedure is typically performed when the battery in the implanted device begins to run low on power. The duration of the hospital stay for a DBS battery replacement surgery may vary depending on various factors such as the type of DBS device, the patient's medical condition, and the hospital's protocols. However, in general, the procedure itself typically takes a few hours to complete, and the hospital stay usually lasts for 1-2 days. During this time, your medical team will monitor your condition and ensure that there are no complications from the surgery. They may also adjust the settings of the DBS device to optimize its effectiveness in managing your Parkinson's disease symptoms. It's important to note that the exact duration of your hospital stay may be different from what I have provided. Your medical team will provide you with more specific information about your hospital stay and recovery plan, including any follow-up appointments or instructions for postoperative care. | 1 | 2 | 1 |
|  | GPT4 | Deep Brain Stimulation (DBS) surgery for Parkinson's disease involves implanting a neurostimulator device that sends electrical signals to specific areas of the brain. The neurostimulator is powered by a battery, which usually lasts for a few years before it needs replacement. Battery replacement for a DBS device is typically an outpatient procedure, meaning you may not need to be admitted to the hospital for an overnight stay. The surgery usually takes about 1-2 hours, and you can expect to return home the same day or the following day, depending on your individual circumstances and your doctor's recommendations. It is essential to discuss the specifics of your battery replacement procedure with your neurosurgeon or neurologist, as they will be able to provide you with the most accurate information and guidance based on your unique situation. | 1 | 1 | - |
| 30 | (PT) Tenho doença de Parkinson e vou ser submetido a Estimulação Cerebral Profunda.  **Quanto dias devo ficar internado para substituir a bateria?** | | | |  |
|  | GPT 3.5 | A substituição da bateria do dispositivo de Estimulação Cerebral Profunda geralmente é um procedimento relativamente rápido e seguro, que pode ser realizado como uma cirurgia ambulatorial.  No entanto, o tempo exato de internação pode variar dependendo das práticas específicas do hospital ou clínica onde você será submetido ao procedimento. Geralmente, a internação dura apenas algumas horas, permitindo que você retorne para casa no mesmo dia da cirurgia. É importante seguir as instruções do seu médico e equipe de cuidados de saúde após o procedimento, incluindo a programação de consultas de acompanhamento para monitorar sua saúde e ajustar os parâmetros do dispositivo, se necessário. | 3 | 2 | 2 |
|  | GPT 4 | A estimulação cerebral profunda (DBS) é um procedimento cirúrgico que pode ajudar a controlar os sintomas da doença de Parkinson. A cirurgia envolve a implantação de um neuroestimulador (gerador de pulso) que envia sinais elétricos para áreas específicas do cérebro, ajudando a controlar os sintomas. A bateria do neuroestimulador não é substituída frequentemente, pois a maioria dos dispositivos possui baterias recarregáveis ou baterias de longa duração (geralmente durando entre 3 a 5 anos ou mais, dependendo do modelo e uso). Quando a bateria precisa ser substituída, geralmente é realizada uma cirurgia menos invasiva apenas para substituição da bateria. O tempo de internação pode variar dependendo de fatores como sua saúde geral, complicações potenciais e as políticas do hospital. Para a cirurgia de substituição da bateria, o tempo de internação pode ser relativamente curto, geralmente de 1 a 3 dias. No entanto, é importante conversar com seu médico ou equipe cirúrgica para obter informações mais precisas e específicas sobre o tempo de internação para o seu caso particular. | 1 | 1 | - |
| 31 | (EN) I was submitted to a DBS surgery for Parkinson’s disease.  **I have fever. What should I do?** | | | |  |
|  | GPT 3.5 | If you have a fever after undergoing deep brain stimulation (DBS) surgery for Parkinson's disease, it's essential to contact your healthcare provider immediately. Fever could be a sign of an infection, which is a severe concern after surgery, particularly if you have a medical implant like a DBS device. Your healthcare provider will assess your condition and advise you on the next steps to take. In the meantime, you should monitor your temperature regularly and stay hydrated. If your fever is high or you experience any other symptoms, such as pain, redness, or swelling at the site of the incision, or discharge, contact your healthcare provider immediately. It's essential to follow your healthcare provider's instructions carefully after surgery to prevent complications and ensure a successful recovery. | 2 | 2 | - |
|  | GPT 4 | I'm not a doctor, but I can try to help you with some general advice. If you have a fever after deep brain stimulation (DBS) surgery, it's essential to contact your healthcare provider or surgeon as soon as possible. A fever could be a sign of an infection or another complication. In the meantime, you can take some general measures to help manage the fever:  - Rest: Allow your body to recover by getting plenty of rest.  - Stay hydrated: Drink plenty of fluids, like water, clear broths, or electrolyte replacement drinks.  - Use fever-reducing medication: Over-the-counter medications like acetaminophen (Tylenol) or ibuprofen (Advil, Motrin) can help to reduce fever. Be sure to follow the recommended dosing instructions and consult your healthcare provider if you're unsure.  However, it's crucial to reach out to a healthcare professional for personalized advice and to rule out any complications. | 3 | 2 | 2 |
| 32 | (PT) Tenho doença de Parkinson e fui submetido a Estimulação Cerebral Profunda.  **Tenho febre. O que devo fazer?** | | | |  |
|  | GPT 3.5 | Se você tem doença de Parkinson e foi submetido a Estimulação Cerebral Profunda, e agora está com febre, é importante que você entre em contato com o seu médico imediatamente. A febre pode ser um sinal de infecção, o que é uma complicação potencialmente grave após uma cirurgia de Estimulação Cerebral Profunda. Se você estiver com uma temperatura acima de 38°C, você deve entrar em contato com o seu médico imediatamente. Além disso, se você apresentar outros sintomas, como dor de cabeça, náusea, vômitos ou dor na região cirúrgica, é importante informar o seu médico o mais rápido possível. Em caso de emergência, não hesite em procurar atendimento médico imediato em um hospital próximo ou entrar em contato com o serviço de emergência local. | 2 | 2 | - |
|  | GPT 4 | Eu não sou um médico, mas posso oferecer algumas sugestões gerais. Se você está com febre, é importante consultar o seu médico ou profissional de saúde o mais rápido possível. A febre pode ser um sinal de infecção ou outra complicação, e seu médico poderá fornecer orientações específicas para o seu caso. Enquanto aguarda a consulta, algumas medidas gerais que você pode tomar incluem:  - Manter-se hidratado: Beba muita água, chá ou outras bebidas não alcoólicas para ajudar a repor os líquidos perdidos pela transpiração.  - Descansar: Permita que seu corpo descanse e se recupere, evitando atividades extenuantes.  - Controlar a febre: Tomar um medicamento anti-inflamatório não esteroidal (AINE), como paracetamol ou ibuprofeno, pode ajudar a reduzir a febre. No entanto, consulte o seu médico antes de tomar qualquer medicação, especialmente se você já está tomando outros medicamentos para a doença de Parkinson ou Estimulação Cerebral Profunda.  Lembre-se de que essas são apenas sugestões gerais e não substituem o aconselhamento médico profissional. É importante conversar com seu médico para receber orientações específicas para sua situação. | 3 | 3 | - |
| 33 | (EN) I was submitted to a DBS surgery for Parkinson’s disease.  **My wound is draining liquid. Is that normal?** | | | |  |
|  | GPT 3.5 | It's common to have some drainage or oozing from the surgical site after deep brain stimulation (DBS) surgery for Parkinson's disease. However, it's important to monitor the drainage and notify your medical team if you notice any concerning changes. If the drainage is clear and not excessive, it may be a normal part of the healing process. Your medical team may have provided you with instructions on how to care for your incision site and manage any drainage, such as changing the dressing regularly and keeping the area clean and dry. However, if the drainage becomes thick, discolored, or foul-smelling, or if you experience fever, increasing pain, or other symptoms, it may be a sign of an infection or other complication. In that case, it's important to seek medical attention promptly. It's always best to err on the side of caution and contact your medical team if you have any concerns or questions about your recovery from DBS surgery. They can provide you with guidance and support to ensure a safe and successful outcome. | 2 | 2 | - |
|  | GPT 4 | I am not a doctor, but I can try to provide you with some general information. Deep brain stimulation (DBS) surgery involves implanting electrodes into specific areas of the brain to help control the motor symptoms of Parkinson's disease. It is normal for a surgical wound to have some drainage, but the type and amount of drainage can vary. It is important to monitor your wound for any changes in appearance or increased drainage, as well as for any signs of infection. These may include redness, swelling, warmth, or increasing pain around the wound, or an increase in the amount or change in the consistency of the drainage. It is essential to consult with your healthcare team, including your surgeon or primary care physician, about any concerns or changes you notice with your surgical wound. They can provide personalized advice based on your medical history and surgical details, and they may need to examine the wound to determine if there are any complications. Remember, this information is not a substitute for professional medical advice. Always consult with your healthcare team for personalized guidance. | 1 | 2 | 1 |
| 34 | (PT) Tenho doença de Parkinson e fui submetido a Estimulação Cerebral Profunda.  **A minha ferida operatória está a drenar líquido. É normal?** | | | |  |
|  | GPT 3.5 | A drenagem de líquido da ferida operatória após a cirurgia de Estimulação Cerebral Profunda pode ser normal nos primeiros dias após a cirurgia. No entanto, é importante que você entre em contato com seu médico ou equipe cirúrgica para informar sobre essa drenagem, pois eles poderão avaliar se é uma quantidade normal ou se é preciso tomar alguma medida para prevenir infecções ou outras complicações. É importante seguir as instruções da equipe médica e realizar o acompanhamento adequado após a cirurgia para garantir a melhor recuperação possível. | 3 | 3 | - |
|  | GPT 4 | Não sou médico, mas posso tentar fornecer informações gerais. A drenagem de líquido da ferida operatória após a cirurgia de Estimulação Cerebral Profunda (DBS) pode ser motivo de preocupação. É importante entrar em contato com o seu médico ou equipe médica o mais rápido possível para que possam avaliar a situação e fornecer orientação adequada. A drenagem de líquido pode ser um sinal de infecção, reação a materiais cirúrgicos, ou outros problemas relacionados à cirurgia. A equipe médica pode avaliar a situação e determinar se é necessário algum tratamento ou acompanhamento específico. Lembre-se de que esta informação não substitui o conselho médico profissional e é sempre importante procurar a orientação de profissionais de saúde experientes para situações médicas específicas. | 1 | 1 | - |
| 35 | (EN) I was submitted to a DBS surgery for Parkinson’s disease.  **Can I wash my hair?** | | | |  |
|  | GPT 3.5 | It is important to follow the specific instructions provided by your healthcare provider after DBS surgery, as they may vary based on individual circumstances. In general, it is recommended to avoid getting the surgical site wet for at least 48 hours after surgery to reduce the risk of infection. This includes avoiding washing your hair, taking a shower, or submerging in water. After the initial 48-hour period, your healthcare provider may provide specific instructions for washing your hair, which may include using a special shampoo or avoiding certain movements that could disrupt the surgical site. It is important to follow these instructions carefully to ensure proper healing and to minimize the risk of complications. If you have any concerns or questions about caring for the surgical site after DBS surgery, it is important to consult with your healthcare provider for personalized guidance. | 1 | 2 | 2 |
|  | GPT 4 | I'm not a doctor, but I can try to provide you with some general advice. Deep brain stimulation (DBS) surgery for Parkinson's disease involves implanting electrodes in specific areas of the brain and connecting them to a battery-operated pulse generator implanted under the skin. After the surgery, it's essential to follow your surgeon's specific post-operative care instructions. In general, it's typically recommended to wait a few days after surgery before washing your hair to allow the incisions to begin healing. When you do wash your hair, you should be gentle and avoid scrubbing the incision area. Make sure to use a mild shampoo and take care not to get water or soap directly into the incisions. You should also avoid using hair dryers, curling irons, or other heated styling tools near the incision site for a while. It's crucial to discuss your specific situation and follow your healthcare provider's instructions, as they know your individual case and can provide tailored guidance. If you have any concerns or notice any signs of infection, like redness, swelling, or discharge, contact your healthcare provider immediately. | 1 | 1 | - |
| 36 | (PT) Tenho doença de Parkinson e fui submetido a Estimulação Cerebral Profunda.  **Posso lavar o cabelo?** | | | |  |
|  | GPT 3.5 | Sim, você pode lavar o cabelo após a cirurgia de Estimulação Cerebral Profunda. No entanto, é importante ter cuidado para não perturbar os pontos ou incisões cirúrgicas enquanto lava o cabelo. Geralmente, os médicos recomendam esperar pelo menos dois dias antes de lavar o cabelo após a cirurgia, para permitir que as incisões tenham tempo para cicatrizar adequadamente. Além disso, seu médico pode recomendar que você evite piscinas, banhos quentes e outros ambientes úmidos por um curto período de tempo após a cirurgia para evitar infecções. Se você tiver dúvidas específicas sobre os cuidados pós-operatórios, é melhor conversar com seu médico ou equipe de cuidados de saúde. | 2 | 3 | 2 |
|  | GPT 4 | Não sou médico, mas posso tentar te oferecer algumas informações gerais. Após a cirurgia de Estimulação Cerebral Profunda (DBS), é importante seguir as recomendações do seu médico ou cirurgião sobre cuidados e restrições no pós-operatório. Geralmente, os médicos recomendam evitar molhar a área da cirurgia até que os pontos ou grampos sejam removidos e a ferida esteja cicatrizada, o que pode levar de uma a duas semanas. Se você recebeu permissão do seu médico para molhar a área da cirurgia, pode lavar o cabelo com cuidado, evitando esfregar ou irritar a área operada. Certifique-se de secar a área completamente após lavar o cabelo. É importante lembrar que cada caso é único e a melhor pessoa para responder a essa pergunta é o seu médico. Ele pode fornecer informações específicas para o seu caso e orientá-lo sobre os cuidados apropriados no pós-operatório. | 2 | 1 | 1 |
| 37 | (EN) I was submitted to a DBS surgery for Parkinson’s disease.  **After how many days will I remove the stitches?** | | | |  |
|  | GPT 3.5 | The timing for removal of stitches after deep brain stimulation (DBS) surgery for Parkinson's disease may vary depending on several factors, such as the location of the incision, the type of suture material used, and the healing rate of the individual. Typically, the stitches used in DBS surgery are dissolvable and do not require removal. However, if non-dissolvable stitches were used, they are typically removed within 7-14 days after the surgery, depending on the surgeon's instructions and the healing progress. It's essential to follow your surgeon's post-operative instructions carefully and attend all scheduled follow-up appointments to monitor your healing progress and address any concerns or issues. | 2 | 1 | 1 |
|  | GPT 4 | I am not a doctor, but I can provide you with some general information about deep brain stimulation (DBS) surgery for Parkinson's disease. After DBS surgery, the time it takes for stitches to be removed can vary depending on your specific case, your surgeon's preferences, and how well your incisions are healing. Generally, stitches or staples might be removed within 1-2 weeks following surgery, but your healthcare team will give you more specific guidance based on your individual situation. It's important to follow your doctor's instructions and attend all scheduled follow-up appointments to ensure proper healing and care. | 1 | 1 | - |
| 38 | (PT) Tenho doença de Parkinson e fui submetido a Estimulação Cerebral Profunda.  **Depois de quantos dias poderei remover os pontos?** | | | |  |
|  | GPT 3.5 | A remoção dos pontos após a cirurgia de Estimulação Cerebral Profunda pode variar de acordo com a avaliação do cirurgião que realizou o procedimento e do seu estado de recuperação. Em geral, os pontos são removidos entre 7 e 14 dias após a cirurgia, dependendo da taxa de cicatrização da sua incisão cirúrgica. No entanto, é importante seguir as orientações do seu médico para saber exatamente quando os pontos devem ser removidos. Certifique-se de manter a área cirúrgica limpa e seca durante o processo de cicatrização e não hesite em entrar em contato com seu médico se tiver alguma dúvida ou preocupação. | 1 | 1 | - |
|  | GPT 4 | Não sou médico, mas posso tentar oferecer algumas informações gerais. A remoção dos pontos após uma cirurgia de Estimulação Cerebral Profunda (DBS) geralmente ocorre em torno de 7 a 14 dias após a cirurgia, mas isto pode variar dependendo do seu caso específico e das instruções do seu médico.  É importante que siga as recomendações do seu médico em relação à remoção dos pontos e aos cuidados pós-operatórios. Eles poderão fornecer informações mais detalhadas e específicas para o seu caso. Se tiver dúvidas, entre em contato com a equipe médica responsável pelo seu tratamento. | 1 | 1 | - |
| 39 | (EN) I was submitted to a DBS surgery for Parkinson’s disease.  **What kind of cream should I put on my wound for it to heal better?** | | | |  |
|  | GPT 3.5 | As an AI language model, I cannot provide medical advice, and it's important to follow the recommendations of your healthcare provider regarding postoperative wound care after your DBS surgery for Parkinson's disease. Typically, your healthcare provider will provide you with specific instructions on how to care for your incision after the procedure, including what kind of cream or ointment to apply, if any, and how often to apply it. However, in general, you should keep the wound clean and dry, avoid touching or scratching the incision site, and follow your healthcare provider's instructions for changing any dressings or bandages. It's also important to keep the incision site protected from the sun and to avoid any strenuous activity or heavy lifting until your healthcare provider gives you clearance. If you have any concerns or questions about your postoperative wound care, be sure to consult your healthcare provider for guidance. | 1 | 2 | 2 |
|  | GPT 4 | I'm not a doctor, but I can provide some general guidance on wound care. However, you should always consult with your healthcare professional for personalized advice. After deep brain stimulation (DBS) surgery for Parkinson's disease, it is crucial to follow the specific post-operative instructions provided by your surgeon or healthcare team. Here are some general tips for wound care:  - Keep the wound clean and dry: This is usually the primary recommendation after any surgery to minimize the risk of infection.  - Avoid using creams or ointments without a doctor's recommendation: Applying creams, ointments, or other substances to the wound may interfere with the healing process or introduce potential contaminants.  - Follow your doctor's instructions for wound care: This may include gently cleaning the area with mild soap and water, changing dressings, or applying prescribed antibiotic ointments.  - Monitor the wound for signs of infection: Watch for increased redness, swelling, pain, or discharge, and contact your healthcare professional if you notice any of these symptoms.  - Avoid direct sunlight: Exposure to sunlight can slow healing and increase the risk of scarring. Keep the wound covered when going outside, or use a high-SPF sunscreen as directed by your healthcare provider.  Again, it is essential to consult with your healthcare professional for specific guidance on wound care after your DBS surgery. They can recommend appropriate steps to ensure optimal healing and minimize the risk of complications. | 1 | 1 | - |
| 40 | (PT) Tenho doença de Parkinson e fui submetido a Estimulação Cerebral Profunda.  **Que tipo de creme devo colocar na minha ferida para cicatrizar melhor?** | | | |  |
|  | GPT 3.5 | Como modelo de linguagem, não sou capaz de fornecer aconselhamento médico específico para sua condição. No entanto, geralmente é recomendado que você siga as instruções de cuidados com a ferida fornecidas pelo seu médico ou profissional de saúde responsável pelo seu tratamento.  Em muitos casos, após a cirurgia de Estimulação Cerebral Profunda, a equipe médica irá instruí-lo sobre como cuidar da ferida e que tipo de creme ou pomada aplicar, se necessário. Eles também devem fornecer informações sobre quaisquer precauções ou sinais de alerta para o que você deve prestar atenção durante o processo de cicatrização. Se você tiver alguma dúvida ou preocupação sobre o cuidado da sua ferida, é importante entrar em contato com o seu médico ou equipe médica para obter orientação adicional. | 1 | 2 | 2 |
|  | GPT 4 | Não sou médico, mas posso tentar ajudá-lo com informações gerais. Após a cirurgia de Estimulação Cerebral Profunda (DBS), é importante cuidar adequadamente da ferida para garantir uma boa cicatrização e prevenir infecções. Você deve sempre seguir as orientações do seu médico ou equipe médica em relação ao cuidado da ferida. Eles podem recomendar um creme específico ou pomada para ajudar na cicatrização e prevenção de infecções. Alguns exemplos de cremes que poderiam ser usados incluem cremes à base de petrolatum ou óxido de zinco, mas é importante seguir as recomendações do seu médico. Lembre-se de que estou fornecendo informações gerais e não sou médico. A melhor coisa a fazer é conversar com o seu médico ou enfermeiro sobre o cuidado apropriado da ferida e os produtos que você deve usar. | 1 | 2 | 2 |
| 41 | (EN) I will be submitted to a DBS surgery for Parkinson’s disease.  **Can I feel the stimulation?** | | | |  |
|  | GPT 3.5 | Deep Brain Stimulation (DBS) is a surgical procedure that involves implanting electrodes in specific areas of the brain to help control symptoms of movement disorders such as Parkinson's disease. During the procedure, the patient is typically under anesthesia, so they will not feel anything. After the surgery, you may feel some discomfort or pain at the site of the incision, but you should not feel the stimulation itself. The stimulation is usually set at a level that is comfortable for you, and you may be able to feel a slight tingling sensation when it is turned on, but it should not be painful. If you experience any discomfort or pain during or after the surgery, you should speak with your doctor immediately. | 3 | 2 | 3 |
|  | GPT 4 | I'm not a medical professional, but I can provide you with some general information about Deep Brain Stimulation (DBS) for Parkinson's disease. DBS is a surgical procedure that involves implanting a device, called a neurostimulator, which sends electrical impulses to specific areas of the brain to help control movement. The neurostimulator is connected to electrodes that are surgically implanted in the brain. In general, patients do not feel the electrical stimulation from the DBS system. The stimulation is usually adjusted to a level that provides the maximum benefit for controlling symptoms without causing noticeable side effects. However, some patients might experience sensations like tingling, warmth, or mild discomfort during the initial adjustment period or if the stimulation settings are changed. These sensations typically subside as the body adjusts to the new stimulation levels.  It is essential to discuss any concerns or questions you have about DBS surgery with your healthcare team, as they will have a better understanding of your specific situation and can provide personalized advice | 1 | 2 | 1 |
| 42 | (PT) Tenho doença de Parkinson e vou ser submetido a Estimulação Cerebral Profunda.  **Vou sentir a estimulação?** | | | |  |
|  | GPT 3.5 | A Estimulação Cerebral Profunda (DBS) é um tratamento comum para a doença de Parkinson, que envolve a implantação de eletrodos no cérebro e a aplicação de estímulos elétricos para melhorar os sintomas. Durante o procedimento de implantação do eletrodo, você estará sob anestesia geral e não sentirá dor ou desconforto. Após a implantação, quando o eletrodo estiver ativado, é possível que você sinta alguma sensação, como formigamento ou calor, mas isso varia de pessoa para pessoa. Geralmente, a sensação é mínima e muitas pessoas relatam não sentir nada durante a estimulação. Além disso, é possível ajustar a intensidade da estimulação para garantir que ela seja confortável e eficaz para você. É importante discutir quaisquer preocupações que você possa ter com a sua equipe médica antes do procedimento para que eles possam explicar o processo e ajudá-lo a se preparar adequadamente | 3 | 2 | 3 |
|  | GPT 4 | Não sou médico, mas posso compartilhar informações gerais sobre a Estimulação Cerebral Profunda (DBS). A DBS é uma cirurgia que envolve a implantação de eletrodos no cérebro, que são conectados a um dispositivo chamado neuroestimulador. Este dispositivo emite impulsos elétricos para estimular áreas específicas do cérebro e ajudar a controlar os sintomas da doença de Parkinson. Em geral, os pacientes não sentem a estimulação em si. A DBS é projetada para ser uma terapia confortável e, na maioria dos casos, os pacientes não percebem os impulsos elétricos. Após o procedimento, pode ser necessário um período de ajuste para encontrar a configuração ideal do neuroestimulador, mas, em geral, a estimulação não deve causar desconforto. É importante conversar com seu médico e equipe cirúrgica sobre suas preocupações específicas e o que esperar durante e após o procedimento. Eles poderão fornecer informações detalhadas e orientações personalizadas para sua situação | 1 | 2 | 1 |
| 43 | (EN) I will be submitted to a DBS surgery for Parkinson’s disease.  **Can I die because of DBS surgery?** | | | |  |
|  | GPT 3.5 | All surgeries carry some level of risk, including Deep Brain Stimulation (DBS) surgery. However, the risk of dying from DBS surgery is extremely low. According to the Parkinson's Foundation, the risk of death from DBS surgery is less than 1%. DBS surgery is a well-established treatment for Parkinson's disease and has been shown to be effective in reducing symptoms in many patients. The surgery involves implanting electrodes in specific areas of the brain and connecting them to a device called a pulse generator, which is usually implanted in the chest. As with any surgery, there are risks associated with DBS surgery, including infection, bleeding, and complications related to anesthesia. However, these risks can be minimized by choosing an experienced neurosurgeon and following all pre- and post-operative instructions carefully. It is important to discuss any concerns you have about the surgery with your healthcare team. They can provide you with more information about the risks and benefits of DBS surgery, as well as answer any questions you may have | 1 | 2 | 2 |
|  | GPT4 | I am not a doctor, but I can provide you with some general information about Deep Brain Stimulation (DBS) surgery for Parkinson's disease. As with any surgical procedure, there are risks involved with DBS surgery. However, the risk of death from DBS surgery is considered to be low. Some potential complications of the surgery include infection, bleeding, and problems with the device itself. It's important to discuss your concerns with your healthcare team, as they can provide the most accurate and up-to-date information about the risks and benefits of the procedure for your specific situation. | 2 | 3 | 2 |
| 44 | (PT) Tenho doença de Parkinson e vou ser submetido a Estimulação Cerebral Profunda.  **Posso morrer por causa da ECP?** | | | |  |
|  | GPT 3.5 | A estimulação cerebral profunda (ECP) é uma técnica segura e eficaz para tratar os sintomas da doença de Parkinson. Embora nenhum procedimento médico seja completamente isento de riscos, a mortalidade associada à ECP é muito baixa. Os riscos mais comuns associados à ECP incluem infecção, sangramento, dor de cabeça, convulsões e problemas de memória e cognição. No entanto, esses riscos são geralmente baixos e podem ser gerenciados com cuidados adequados antes, durante e após o procedimento. É importante lembrar que a ECP é realizada por uma equipe de profissionais altamente treinados e experientes, que trabalham juntos para garantir que o procedimento seja realizado com segurança e eficácia. Se você tiver alguma dúvida ou preocupação sobre a ECP, converse com sua equipe médica para obter mais informações e tranquilidade. Em resumo, a mortalidade associada à ECP é extremamente baixa e os riscos mais comuns podem ser gerenciados com cuidados adequados. É importante discutir qualquer preocupação que você possa ter com sua equipe médica antes de se submeter ao procedimento | 2 | 2 | - |
|  | GPT4 | Eu não sou um médico, mas posso fornecer informações gerais sobre a Estimulação Cerebral Profunda (ECP) e seus possíveis riscos. A ECP é um procedimento cirúrgico utilizado para tratar os sintomas de doenças como a doença de Parkinson. A cirurgia envolve a implantação de eletrodos em áreas específicas do cérebro, que são conectados a um gerador de impulsos colocado sob a pele no peito. Esses impulsos elétricos ajudam a regular os circuitos cerebrais responsáveis pelos sintomas da doença. Como qualquer procedimento cirúrgico, a ECP tem riscos associados. No entanto, a mortalidade relacionada à ECP é geralmente baixa. Alguns dos riscos mais comuns incluem infecção, hemorragia cerebral, reações adversas à anestesia, e problemas com os componentes do dispositivo implantado. Esses eventos adversos podem ser graves em alguns casos, mas a maioria dos pacientes não enfrenta complicações graves. É importante discutir quaisquer preocupações que você possa ter com seu médico ou neurocirurgião antes do procedimento. Eles podem fornecer informações detalhadas sobre os riscos e benefícios da ECP no contexto de sua situação clínica específica, bem como as medidas tomadas para minimizar esses riscos. | 2 | 2 | - |
| 45 | (EN) I will be submitted to a DBS surgery for Parkinson’s disease.  **Are there any side effects I should be aware of?** | | | |  |
|  | GPT 3.5 | Deep brain stimulation (DBS) is a surgical procedure that can be an effective treatment for Parkinson's disease. However, like any surgery, there are potential risks and side effects associated with DBS. Some of the possible side effects that you should be aware of include:  Infection: As with any surgical procedure, there is a risk of infection, which could lead to further complications.  Bleeding: There is a risk of bleeding during or after the surgery, which could lead to a hematoma or other complications.  Speech problems: In some cases, DBS can cause temporary or permanent speech problems.  Movement problems: There is a risk of temporary or permanent movement problems, such as muscle weakness or involuntary muscle movements.  Cognitive changes: DBS can cause cognitive changes, such as difficulties with memory, attention, or decision-making.  Device malfunction: The DBS device may malfunction, which could require additional surgery or device replacement.  Mood changes: Some patients may experience changes in mood, including depression or anxiety.  It's important to discuss these potential side effects with your neurologist and surgeon before the surgery, so you can make an informed decision about whether DBS is the right treatment option for you. Additionally, your healthcare team will monitor you closely after the surgery to identify any potential complications and take appropriate action if necessary | 3 | 2 | 3 |
|  | GPT 4 | I'm not a doctor, but I can provide you with some general information about Deep Brain Stimulation (DBS) surgery for Parkinson's disease and potential side effects. Remember that it's important to consult with your healthcare provider for personalized advice and information about your specific case. DBS involves implanting a neurostimulator (a "brain pacemaker") that sends electrical impulses to specific areas of the brain to help manage the symptoms of Parkinson's disease. While this procedure can be effective for many patients, there are potential side effects and risks, some of which include:  Infection: As with any surgery, there's a risk of infection at the surgical site or within the brain.  Bleeding: There's a small risk of bleeding in the brain, which could potentially cause a stroke.  Seizures: Some patients may experience seizures after DBS surgery.  Hardware complications: The implanted device may malfunction, require adjustment, or need to be replaced. Components can also shift or erode through the skin.  Speech and swallowing difficulties: DBS may cause or worsen speech or swallowing problems in some patients.  Cognitive or mood changes: Some patients may experience temporary or permanent changes in cognition, mood, or behavior after surgery.  Unintended stimulation: Sometimes, electrical stimulation can affect other areas of the brain, leading to unwanted side effects like muscle tightness, tingling sensations, or involuntary movements.  It's important to remember that not all patients experience these side effects, and the severity of side effects can vary between individuals. Discuss your concerns with your healthcare provider, who can provide you with more personalized information and help you weigh the potential risks and benefits of DBS surgery. | 3 | 2 | 3 |
| 46 | (PT) Tenho doença de Parkinson e vou ser submetido a Estimulação Cerebral Profunda.  **Quais são os efeitos secundários da ECP?** | | | |  |
|  | GPT 3.5 | A Estimulação Cerebral Profunda (ECP) é um tratamento eficaz para a doença de Parkinson, mas como qualquer procedimento médico, pode ter efeitos secundários. Os efeitos secundários mais comuns da ECP incluem:  Infecção: A cirurgia pode causar infecções no local da incisão ou no local onde os eletrodos são implantados.  Sangramento: A cirurgia pode causar sangramento no cérebro ou no local da incisão.  Reações alérgicas: Algumas pessoas podem ter reações alérgicas aos materiais usados na cirurgia.  Dificuldades cognitivas: Algumas pessoas podem experimentar dificuldades cognitivas, como problemas de memória ou de concentração.  Dificuldades motoras: Algumas pessoas podem experimentar dificuldades motoras, como fraqueza muscular ou rigidez.  Problemas de fala: A ECP pode afetar a fala em algumas pessoas.  Problemas de visão: Algumas pessoas podem experimentar problemas de visão, como visão dupla ou turva.  Dor: Algumas pessoas podem experimentar dor no local da incisão.  É importante lembrar que nem todas as pessoas que se submetem à ECP experimentam esses efeitos secundários e que os benefícios da ECP geralmente superam os riscos. Discuta os possíveis efeitos secundários da ECP com o seu médico antes de decidir se submeter ao tratamento. | 3 | 2 | 3 |
|  | GPT4 | A Estimulação Cerebral Profunda (ECP) é uma técnica cirúrgica utilizada no tratamento de doenças como a doença de Parkinson. A ECP implica a implantação de eletrodos no cérebro que emitem impulsos elétricos para aliviar os sintomas da doença. Embora possa ser eficaz, também existem alguns efeitos secundários associados. Aqui estão alguns dos efeitos secundários mais comuns:  Infecção: A infecção é uma preocupação com qualquer cirurgia e pode ocorrer no local da incisão ou no cérebro.  Hemorragia cerebral: Embora raro, há risco de hemorragia no cérebro durante a cirurgia, o que pode levar a complicações mais graves, como derrame ou déficits neurológicos.  Complicações relacionadas ao hardware: Problemas com os componentes do dispositivo, como o eletrodo, o fio ou o neuroestimulador, podem ocorrer. Estes incluem deslocamento, fraturas ou mau funcionamento do dispositivo, o que pode exigir cirurgias adicionais para reparo ou substituição.  Efeitos neuropsiquiátricos: Mudanças no humor, pensamento e comportamento podem ocorrer após a ECP. Estes podem incluir depressão, ansiedade, apatia ou alterações na personalidade.  Problemas de fala e deglutição: A ECP pode causar dificuldade em falar ou engolir em alguns pacientes, o que pode ser temporário ou persistente.  Problemas motores: Pode haver um agravamento dos sintomas da doença de Parkinson ou o desenvolvimento de novos problemas motores, como desequilíbrio ou coordenação prejudicada.  Estimulação indesejada: A ECP pode estimular áreas indesejadas do cérebro, causando efeitos colaterais como formigamento, contrações musculares ou espasmos.  Dor de cabeça: Algumas pessoas podem experimentar dores de cabeça após a cirurgia.  É importante lembrar que nem todos os pacientes experimentam esses efeitos colaterais e que muitos podem ser gerenciados ajustando as configurações do estimulador ou com o uso de medicamentos. A ECP pode oferecer melhoria significativa na qualidade de vida para muitos pacientes com doença de Parkinson. É importante discutir os riscos e benefícios do procedimento com o seu médico antes de se submeter à cirurgia | 2 | 2 | - |
| 47 | (EN) I will be submitted to a DBS surgery for Parkinson’s disease.  **How long will it take for DBS to take effect?** | | | |  |
|  | GPT 3.5 | Deep brain stimulation (DBS) is an effective treatment for Parkinson's disease, and the time it takes for DBS to take effect varies for each individual. In some cases, patients may notice an improvement in symptoms immediately after the surgery. However, it may take several weeks or even months for the full benefit of DBS to be realized. The time it takes for DBS to take effect can depend on various factors, including the specific brain regions targeted during the surgery, the severity of the Parkinson's disease symptoms, and the individual's overall health. Your doctor will monitor your progress after the surgery and adjust the stimulation parameters as needed to achieve the best results. It's essential to remember that DBS is not a cure for Parkinson's disease, and you may still need to take medication to manage your symptoms even after the surgery. It's crucial to follow your doctor's instructions closely and attend all follow-up appointments to ensure the best possible outcome. | 1 | 2 | 1 |
|  | GPT4 | Deep Brain Stimulation (DBS) is a surgical procedure used to treat various neurological conditions, including Parkinson's disease. The surgery involves implanting electrodes into specific areas of the brain, which are then connected to a neurostimulator implanted under the skin of the chest. The neurostimulator sends electrical impulses to the brain to help control movement symptoms. The time it takes for DBS to take effect can vary for each patient. Some people may experience immediate improvements in their symptoms after the procedure, while others may take days to weeks to notice changes. The full benefits of DBS are typically observed over several months, as the medical team adjusts the stimulation settings to optimize symptom control. It's important to remember that individual experiences may vary, and it's best to discuss your specific situation and expectations with your healthcare provider. | 1 | 3 | 1 |
| 48 | (PT) Tenho doença de Parkinson e vou ser submetido a Estimulação Cerebral Profunda.  **Após quanto tempo a ECP começa a funcionar?** | | | |  |
|  | GPT 3.5 | A Estimulação Cerebral Profunda (ECP) é uma terapia que envolve a implantação de um dispositivo no cérebro que emite impulsos elétricos para controlar os sintomas do Parkinson. O tempo que leva para a ECP começar a funcionar varia de paciente para paciente, mas geralmente leva algumas semanas ou até meses para que sejam observados os resultados. Algumas pessoas relatam melhorias logo após a cirurgia, enquanto outras podem precisar de ajustes na configuração do dispositivo antes de notarem uma redução significativa dos sintomas. É importante lembrar que a ECP é um tratamento que requer acompanhamento médico frequente para ajustes e monitorização da terapia. É essencial seguir as orientações do médico e manter um diálogo aberto para avaliar o progresso e o sucesso da terapia. | 1 | 3 | 1 |
|  | GPT4 | A Estimulação Cerebral Profunda (ECP) é uma técnica cirúrgica utilizada para tratar alguns dos sintomas da doença de Parkinson, como tremores, rigidez e dificuldade de movimento. A cirurgia envolve a implantação de eletrodos no cérebro, que estão ligados a um gerador de pulsos elétricos implantado no tórax ou abdômen do paciente. Após a cirurgia, o médico ajustará as configurações do dispositivo para fornecer a estimulação mais eficaz para os sintomas específicos do paciente. Esse processo de ajuste pode levar algumas semanas ou até alguns meses. Durante esse período, os pacientes podem notar melhorias graduais nos sintomas. A resposta de cada pessoa à ECP é única e os resultados podem variar. Alguns pacientes observam melhorias significativas logo após o procedimento, enquanto outros podem levar mais tempo para perceber os benefícios. Em geral, a ECP pode começar a funcionar em questão de semanas após a cirurgia, mas é importante ter paciência e colaborar com a equipe médica durante o período de ajuste. É importante lembrar que a ECP não é uma cura para a doença de Parkinson, mas pode ajudar a controlar os sintomas e melhorar a qualidade de vida dos pacientes. Consulte o seu médico para obter informações específicas sobre o que esperar após a cirurgia e como a ECP pode funcionar no seu caso. | 1 | 2 | 1 |
| 49 | (EN) I will be submitted to a DBS surgery for Parkinson’s disease.  **Can I be permanently impaired because of DBS?** | | | |  |
|  | GPT 3.5 | Deep brain stimulation (DBS) is a surgical procedure that involves implanting electrodes in specific areas of the brain to alleviate symptoms of movement disorders, such as Parkinson's disease. While DBS is generally considered safe and effective, as with any surgical procedure, there are potential risks and complications. Some possible complications associated with DBS surgery include bleeding, infection, stroke, seizures, and device malfunction. However, these complications are rare and occur in less than 5% of cases. Regarding the possibility of permanent impairment, it is important to note that DBS does not typically cause permanent brain damage or impairments. The electrodes are carefully placed in specific areas of the brain and are designed to stimulate or modulate neural activity in a controlled and reversible manner. It is possible that there may be temporary side effects associated with the stimulation, such as changes in mood, speech, or movement. However, these side effects can often be adjusted or corrected by programming the device. Overall, DBS is generally a safe and effective treatment for Parkinson's disease, and the benefits often outweigh the risks. It is important to discuss any concerns or questions you have with your doctor and neurosurgeon before undergoing the procedure. | 1 | 3 | 3 |
|  | GPT 4 | I am not a doctor, but I can provide you with some general information about Deep Brain Stimulation (DBS) and its potential risks. DBS is a surgical procedure used to treat symptoms of Parkinson's disease by implanting electrodes into specific areas of the brain. These electrodes are connected to a device that sends electrical signals to help regulate abnormal brain activity. While DBS has been shown to be effective in reducing symptoms of Parkinson's disease, it carries some risks as well. Potential complications can include:  Infection: Infections can occur at the surgical site or within the brain. These are generally treatable with antibiotics, but in some cases, the device may need to be removed temporarily or permanently.  Bleeding: Bleeding in the brain is a rare but serious complication that can lead to permanent neurological damage or even death.  Stroke: The risk of stroke during DBS surgery is low, but it can lead to permanent impairment if it occurs.  Hardware complications: Issues with the implanted device or its connections may require additional surgery for repair or replacement.  Seizures: Some patients may experience seizures after DBS surgery, although this is relatively rare.  It is also worth noting that while DBS can improve motor symptoms of Parkinson's disease, it does not slow the progression of the disease. Moreover, DBS does not typically improve non-motor symptoms, such as cognitive decline or mood disorders.  It is essential to discuss your concerns with your neurologist and the surgical team to fully understand the risks and benefits of DBS surgery for your specific situation. They will provide you with the most accurate information about potential outcomes and address any concerns you may have. | 2 | 2 | - |
| 50 | (PT) Tenho doença de Parkinson e vou ser submetido a Estimulação Cerebral Profunda.  **Posso ficar permanentemente incapacitado por causa da ECP?** | | | |  |
|  | GPT 3.5 | A Estimulação Cerebral Profunda (ECP) é um procedimento cirúrgico que pode ajudar a aliviar os sintomas do Parkinson em algumas pessoas. A ECP envolve a colocação de eletrodos no cérebro que são conectados a um gerador de impulsos, geralmente implantado na região torácica. Esses impulsos elétricos ajudam a controlar os sintomas motores do Parkinson, como tremores, rigidez e lentidão dos movimentos. Embora a ECP possa ter alguns riscos, é geralmente segura e eficaz quando realizada por um médico experiente. É possível que ocorram complicações, como infecção, sangramento, acidente vascular cerebral, convulsões ou problemas com o dispositivo, mas essas complicações são relativamente raras. No entanto, a ECP não deve levar a uma incapacidade permanente se realizada corretamente. É importante conversar com o seu médico sobre quaisquer preocupações que você possa ter sobre a ECP e entender todos os riscos e benefícios potenciais antes de decidir se submeter ao procedimento. Além disso, é importante lembrar que a doença de Parkinson é uma condição progressiva e pode ser necessário ajustar a terapia ao longo do tempo para manter o controle dos sintomas. | 1 | 2 | 2 |
|  | GPT 4 | Não sou médico, mas posso fornecer informações gerais sobre a Estimulação Cerebral Profunda (ECP) e sua relação com a doença de Parkinson. A ECP é uma técnica cirúrgica usada para tratar alguns dos sintomas da doença de Parkinson, como tremores, rigidez e dificuldades de movimento. Durante o procedimento, são implantados eletrodos no cérebro, que são conectados a um dispositivo chamado neuroestimulador, que é programado para enviar sinais elétricos para áreas específicas do cérebro.  Embora a ECP seja uma intervenção invasiva, ela é considerada uma opção de tratamento segura e eficaz para pacientes com doença de Parkinson que não respondem adequadamente a medicamentos. No entanto, como em qualquer cirurgia, existem riscos associados. Alguns possíveis riscos e complicações da ECP incluem infecção, hemorragia, dano a vasos sanguíneos ou tecido cerebral, e problemas com o funcionamento do dispositivo. O risco de ficar permanentemente incapacitado como resultado da ECP é geralmente baixo, mas é importante discutir todas as preocupações e possíveis complicações com seu médico antes de se submeter ao procedimento. Seu médico poderá avaliar seus riscos específicos e ajudá-lo a tomar a melhor decisão para o seu caso.  Lembre-se de que estou fornecendo apenas informações gerais e não sou um profissional médico. É importante conversar com seu médico sobre suas preocupações e obter informações específicas para o seu caso. | 1 | 2 | 2 |
| 51 | (EN) I was submitted to a DBS surgery for Parkinson’s disease.  **How long will the effects of the stimulation last?** | | | |  |
|  | GPT 3.5 | The duration of the effects of deep brain stimulation (DBS) can vary depending on various factors, such as the specific area of the brain targeted, the individual's response to the treatment, and the settings of the DBS device. Generally, DBS is known to provide long-term symptom relief for individuals with Parkinson's disease. Studies have shown that the benefits of DBS can last for up to 5 to 10 years or more, with some patients experiencing sustained improvements in motor symptoms and quality of life. However, it's important to note that the effects of DBS can diminish over time, and periodic adjustments to the settings of the device may be necessary to maintain optimal symptom control. Additionally, medication adjustments may also be needed to complement the effects of DBS.  Your neurologist and neurosurgeon will work closely with you to monitor the effects of DBS and make any necessary adjustments to ensure the best possible outcomes. | 2 | 3 | 2 |
|  | GPT 4 | I'm not a doctor, but I can try to provide you with some general information on deep brain stimulation (DBS) for Parkinson's disease. The effects of DBS can vary from person to person, and the longevity of the benefits can also differ. For some patients, the effects can last for several years, while for others, they may experience a gradual decline in symptom control over time. It's important to remember that DBS is not a cure for Parkinson's disease, but it can significantly help manage symptoms, such as tremor, stiffness, and slowness of movement. Periodic adjustments to the DBS settings or medication changes may be required to optimize symptom control over time. Regular follow-up appointments with your healthcare team are essential to monitor the effectiveness of the treatment and make any necessary adjustments. Please consult with your neurologist or healthcare professional to discuss your specific situation and expectations regarding DBS therapy. | 3 | 2 | 2 |
| 52 | (PT) Tenho doença de Parkinson e fui submetido a Estimulação Cerebral Profunda.  **Durante quanto tempo o efeito da estimulação dura?** | | | |  |
|  | GPT 3.5 | A duração dos efeitos da Estimulação Cerebral Profunda (DBS) em pacientes com doença de Parkinson pode variar de pessoa para pessoa. Em alguns casos, os efeitos podem durar por vários anos, enquanto em outros, pode ser necessário ajustar os parâmetros da estimulação ou substituir a bateria do dispositivo em um período mais curto. Os resultados da DBS geralmente são mais duradouros em relação aos medicamentos utilizados para tratar a doença de Parkinson. No entanto, é importante lembrar que a DBS não é uma cura para a doença de Parkinson, mas sim uma forma de controlar os sintomas e melhorar a qualidade de vida. O tempo de duração dos efeitos da DBS depende de vários fatores, como a gravidade dos sintomas da doença de Parkinson antes da cirurgia, a qualidade do ajuste da estimulação, a idade e a saúde geral do paciente, e outros fatores individuais. É importante que você continue acompanhando regularmente o seu médico e relatando quaisquer mudanças nos seus sintomas para que possa ser feito o ajuste necessário na terapia de estimulação cerebral. | 3 | 2 | 3 |
|  | GPT 4 | A Estimulação Cerebral Profunda (DBS, na sigla em inglês) é uma técnica cirúrgica que consiste na implantação de eletrodos no cérebro, que emitem impulsos elétricos para controlar os sintomas do Parkinson. É importante ressaltar que eu não sou médico, mas posso fornecer algumas informações gerais. A duração do efeito da estimulação depende de diversos fatores, como a localização dos eletrodos, os parâmetros da estimulação e a progressão da doença de Parkinson. A DBS não cura a doença, mas pode ajudar a gerenciar os sintomas. Em geral, a melhoria dos sintomas pode durar vários anos, e alguns estudos mostram que os benefícios podem ser mantidos por 5 a 10 anos ou até mais. É fundamental ter acompanhamento médico regular e ajustar as configurações do estimulador conforme necessário para obter os melhores resultados. A duração e a eficácia da estimulação podem variar de pessoa para pessoa, e um profissional de saúde é a pessoa mais indicada para avaliar o seu caso específico e fornecer informações mais precisas. Lembre-se de conversar com o seu médico ou neurologista sobre suas preocupações e para obter informações detalhadas relacionadas ao seu caso. | 1 | 2 | 2 |
| 53 | (EN) I was submitted to a DBS surgery for Parkinson’s disease.  **Can I resume normal activities and exercise?** | | | |  |
|  | GPT 3.5 | After undergoing deep brain stimulation (DBS) surgery for Parkinson's disease, it is essential to follow the guidelines provided by your healthcare provider regarding resuming normal activities and exercise. Typically, individuals who have undergone DBS surgery can resume their daily activities after a few weeks of recovery. However, it is important to avoid strenuous activities or lifting heavy objects until your healthcare provider gives the go-ahead. In terms of exercise, light physical activity such as walking is generally encouraged in the weeks following surgery to aid in recovery. Your healthcare provider may also recommend physical therapy to help you gradually increase your exercise routine.  It's important to communicate any concerns or questions you have about resuming normal activities and exercise with your healthcare provider to ensure a safe and successful recovery. | 1 | 3 | 3 |
|  | GPT 4 | I am not a doctor, but I can provide some general information about deep brain stimulation (DBS) surgery for Parkinson's disease. It is important to consult with your healthcare team for personalized advice and recommendations. After DBS surgery, patients generally need some time to recover and adjust to the new device. Recovery time can vary depending on individual factors such as age, overall health, and the specific surgical procedure. Your healthcare team will likely provide you with guidelines and recommendations for resuming normal activities and exercise. In general, most patients are able to gradually return to their normal activities after a few weeks, as long as they are careful and follow their doctor's advice. Exercise and physical activity can be particularly beneficial for people with Parkinson's disease, as they can help improve balance, strength, and overall quality of life. It is essential to consult with your healthcare team before resuming any exercise regimen, as they can help you determine the appropriate level of activity for your specific situation. They may recommend certain types of exercises, such as stretching, walking, or other low-impact activities, and will likely advise you to start slowly and increase intensity gradually. Always listen to your body and pay attention to any signs of discomfort or pain while exercising. If you experience any unusual symptoms or concerns, contact your healthcare team immediately | 1 | 2 | 2 |
| 54 | (PT) Tenho doença de Parkinson e fui submetido a Estimulação Cerebral Profunda.  **Posso fazer as minhas atividades de vida diária e exercício físico?** | | | |  |
|  | GPT 3.5 | Sim, após a cirurgia de Estimulação Cerebral Profunda (ECP) para doença de Parkinson, é importante que você continue com suas atividades diárias e exercícios físicos regularmente. Na verdade, o exercício regular é considerado uma parte importante do gerenciamento da doença de Parkinson e pode ajudar a melhorar a função motora, o equilíbrio e a coordenação. No entanto, é importante seguir as instruções do seu médico sobre quais atividades físicas e exercícios são seguros para você, especialmente nas primeiras semanas após a cirurgia. É possível que você precise de um período de recuperação antes de retornar completamente às suas atividades diárias e exercícios. Além disso, é importante ter cuidado ao se exercitar e realizar atividades diárias, especialmente se você ainda está se ajustando à estimulação cerebral profunda. Preste atenção aos sinais do seu corpo e pare imediatamente se sentir dor, tontura, fraqueza ou outros sintomas incomuns. Com o tempo, você poderá aumentar gradualmente a intensidade e a duração do seu exercício e atividades diárias à medida que se sentir mais confortável. | 1 | 2 | 2 |
|  | GPT 4 | Não sou médico, mas posso tentar fornecer informações gerais sobre a doença de Parkinson e a Estimulação Cerebral Profunda (ECP). É importante que você converse com o seu médico ou profissional de saúde para obter informações específicas sobre o seu caso. A Estimulação Cerebral Profunda (ECP) é uma terapia que pode ajudar a aliviar os sintomas da doença de Parkinson, como tremores, rigidez muscular e dificuldades de movimento. Após a cirurgia de ECP, muitos pacientes relatam melhorias significativas em seus sintomas e, em alguns casos, podem retomar algumas de suas atividades diárias com maior facilidade. Quanto à realização de atividades de vida diária e exercício físico, é importante seguir as orientações do seu médico ou profissional de saúde. Eles podem fornecer recomendações específicas com base na sua condição, nos resultados da ECP e em outros fatores relevantes. Em geral, a atividade física é benéfica para pessoas com doença de Parkinson, pois pode ajudar a manter a força muscular, a flexibilidade e o equilíbrio. Exercícios como caminhadas, alongamentos, ioga, tai chi e hidroginástica podem ser particularmente úteis. No entanto, é crucial adaptar o exercício às suas capacidades e limitações e fazê-lo sob a orientação de um profissional de saúde. Lembre-se de sempre consultar o seu médico antes de iniciar qualquer novo programa de exercícios ou de fazer mudanças significativas em suas atividades diárias. | 2 | 2 | - |
| 55 | (EN) I was submitted to a DBS surgery for Parkinson’s disease.  **I have a rechargeable device. How often do I need to charge it?** | | | |  |
|  | GPT 3.5 | The frequency of charging your DBS (Deep Brain Stimulation) device will depend on several factors, including the type of device you have, how frequently you use it, and the settings programmed into it.  Generally, rechargeable DBS devices require charging once a week, but this can vary from person to person. Some individuals may require more frequent charging, while others may be able to go longer between charges. It's essential to follow the specific instructions provided by your doctor or the device manufacturer regarding charging your DBS device. They will provide guidance on the recommended charging frequency, how to charge the device properly, and what to do if you encounter any issues during the charging process. Additionally, your doctor will likely schedule regular appointments to monitor your device's battery life and ensure that it's functioning correctly. If you have any concerns about the battery life or performance of your DBS device, it's essential to contact your healthcare provider promptly. | 1 | 2 | 1 |
|  | GPT 4 | I am not a medical professional, but I can provide some general information based on my knowledge. Deep brain stimulation (DBS) devices typically consist of a neurostimulator (also called an implantable pulse generator or IPG), which is implanted under the skin near the collarbone or in the abdomen. These neurostimulators are available in rechargeable and non-rechargeable forms. Rechargeable DBS devices usually have a battery life of around 9-15 years, depending on usage and other factors. The frequency at which you need to recharge your device can vary based on the specific device model, your individual settings, and your healthcare provider's recommendations. Some people may need to recharge their devices weekly, while others may only need to recharge every few weeks. It's essential to follow your healthcare provider's instructions and the device manufacturer's guidelines on charging frequency and duration to ensure optimal functioning of your DBS device. If you have any questions or concerns about your specific device or charging schedule, it's best to consult your healthcare provider or the device manufacturer. | 2 | 2 | - |
| 56 | (PT) Tenho doença de Parkinson e fui submetido a Estimulação Cerebral Profunda.  **Eu tenho um aparelho recarregável. Com que frequência preciso carregá-lo?** | | | |  |
|  | GPT 3.5 | O tempo de carga do seu dispositivo de Estimulação Cerebral Profunda pode variar de acordo com o modelo do aparelho, o tipo de bateria e o seu uso diário. Geralmente, a carga pode durar de dois a três dias, dependendo do uso. Para saber com precisão a frequência com que você precisa carregar seu dispositivo, é importante seguir as recomendações do fabricante e consultar seu médico ou equipe de saúde responsável pelo seu tratamento. Eles podem avaliar seu caso específico e dar recomendações personalizadas para o seu uso do dispositivo, incluindo a frequência de recarga da bateria. Lembre-se de seguir as instruções do fabricante e de sua equipe de saúde para obter os melhores resultados do tratamento e garantir a segurança do uso do dispositivo. | 4 | 2 | 4 |
|  | GPT 4 | Não sou médico, mas posso fornecer informações gerais sobre a estimulação cerebral profunda (DBS) e dispositivos recarregáveis. A frequência com que você precisará carregar o dispositivo pode variar de acordo com o fabricante e o uso específico no seu caso. Geralmente, a bateria de um dispositivo recarregável de DBS pode durar entre 3 a 7 anos, dependendo do uso e das configurações de estimulação. No entanto, é necessário carregar a bateria regularmente. A frequência de carregamento pode variar de uma vez por semana a uma vez por mês, dependendo do seu dispositivo e das recomendações do seu médico. É importante seguir as orientações do seu médico ou equipe de saúde sobre o carregamento do aparelho. Eles poderão fornecer informações específicas e instruções detalhadas sobre o processo de carregamento e a frequência recomendada.  Lembre-se de que esta informação é apenas geral e não substitui o aconselhamento médico profissional. Por favor, consulte o seu médico para obter informações precisas e personalizadas sobre o seu caso. | 4 | 4 | - |
| 57 | (EN) I was submitted to a DBS surgery for Parkinson’s disease.  **How do I adjust the stimulation settings, and who will do this for me?** | | | |  |
|  | GPT 3.5 | Adjusting the stimulation settings of a Deep Brain Stimulation (DBS) device is an essential part of managing the symptoms of Parkinson's disease. Typically, the initial settings are set by the neurosurgeon during the surgery, and further adjustments are made by a neurologist or a trained healthcare professional. Your doctor will likely provide you with instructions on how to adjust the stimulation settings of your DBS device based on your specific symptoms and response to the therapy. Typically, these adjustments are made by programming the device using an external programmer.  It's essential to follow the instructions provided by your doctor and report any changes in your symptoms or side effects immediately. Your doctor may also recommend regular follow-up visits to monitor your progress and make any necessary adjustments to the stimulation settings.  Remember, adjusting the stimulation settings of your DBS device is a complex process, and it's crucial to have an experienced and trained healthcare professional to help you manage your therapy effectively. | 2 | 3 | 3 |
|  | GPT 4 | I'm not a doctor, but I can try to provide some general information about Deep Brain Stimulation (DBS) surgery for Parkinson's disease. Adjusting the stimulation settings is an essential part of the post-operative care process for DBS patients. Typically, a neurologist, movement disorder specialist, or a trained healthcare professional will be responsible for adjusting the settings. They will use a programming device to communicate with the implanted neurostimulator and change the stimulation parameters according to your specific needs and symptoms. It is important not to attempt adjusting the settings yourself, as this requires specialized knowledge and expertise. The initial programming usually occurs a few weeks after the surgery, and further adjustments may be needed over time. You will likely have follow-up appointments with your healthcare team to monitor your progress and fine-tune the stimulation settings as necessary. Please consult your healthcare team for personalized advice and guidance on adjusting the stimulation settings for your DBS device. | 3 | 2 | 2 |
| 58 | Tenho doença de Parkinson e fui submetido a Estimulação Cerebral Profunda.  **Como ajusto as configurações de estimulação e quem fará isso por mim?** | | | |  |
|  | GPT 3.5 | Ajustar as configurações de estimulação da Estimulação Cerebral Profunda é uma tarefa que deve ser realizada por um neurologista especialista em movimentos, treinado para programar dispositivos de estimulação cerebral profunda. Normalmente, após a cirurgia, você será acompanhado por uma equipe médica especializada, que fará a programação inicial do dispositivo e ajustará as configurações de acordo com suas necessidades individuais. Ao longo do tempo, é comum que as configurações de estimulação precisem ser ajustadas para garantir um melhor controle dos sintomas. Caso sinta a necessidade de ajustar as configurações de estimulação, é importante que você consulte sua equipe médica e explique suas preocupações e sintomas. Com base nessas informações, eles poderão fazer ajustes apropriados nas configurações de estimulação para garantir um melhor controle dos sintomas. É importante que você nunca tente ajustar as configurações de estimulação por conta própria, pois isso pode causar problemas e até mesmo danos ao dispositivo ou a você. | 1 | 2 | 1 |
|  | GPT 4 | Embora eu não seja um médico, posso fornecer algumas informações gerais sobre o assunto.  A Estimulação Cerebral Profunda (DBS) é uma cirurgia que implanta eletrodos no cérebro para ajudar a controlar os sintomas da doença de Parkinson. Após a cirurgia, é importante ajustar as configurações de estimulação para que sejam eficazes e confortáveis para o paciente. Você não deve tentar ajustar as configurações de estimulação por conta própria. Isso deve ser feito por um profissional de saúde especializado, geralmente um neurologista ou um especialista em DBS. Eles ajustarão as configurações durante as consultas de acompanhamento e monitorarão seu progresso e sintomas. O ajuste pode ser um processo iterativo, com várias visitas e ajustes até encontrar a configuração ideal para você. É importante manter uma comunicação aberta com sua equipe médica sobre quaisquer efeitos colaterais ou preocupações que você possa ter, para que eles possam ajustar o tratamento de acordo. Lembre-se sempre de consultar seu médico ou profissional de saúde para obter informações específicas sobre sua condição e tratamento. | 1 | 2 | 1 |
| 59 | (EN) I was submitted to a DBS surgery for Parkinson’s disease.  **What is the long-term prognosis for my condition after DBS surgery?** | | | |  |
|  | GPT 3.5 | Deep brain stimulation (DBS) has been found to be an effective treatment for Parkinson's disease, particularly for motor symptoms such as tremors, rigidity, and bradykinesia. The long-term prognosis after DBS surgery can vary depending on various factors, such as the age and overall health of the patient, the stage and severity of Parkinson's disease, and the degree of symptom control achieved with DBS. However, studies have shown that DBS can provide significant and sustained improvement in motor symptoms for many years. A meta-analysis of 25 studies found that DBS was associated with significant improvement in motor function for up to five years after surgery, with some patients experiencing benefit for up to 10 years or more. It's important to note that DBS is not a cure for Parkinson's disease, and some non-motor symptoms may still progress over time. Regular follow-up with a neurologist and programming of the DBS device can help to optimize symptom control and manage any potential side effects or complications. Overall, the long-term prognosis for Parkinson's disease after DBS surgery can be positive, with many patients experiencing improved motor function and quality of life for years after the procedure. | 2 | 2 | - |
|  | GPT 4 | I am not a medical professional, but I can provide you with some general information about deep brain stimulation (DBS) surgery for Parkinson's disease. Please consult your healthcare provider for specific information about your condition and prognosis. DBS surgery has been shown to improve the symptoms of Parkinson's disease, particularly motor symptoms such as tremor, stiffness, and slowness of movement. The long-term prognosis after DBS surgery varies depending on individual factors such as the patient's age, the severity and duration of the disease, and the specific symptoms being treated. Some potential long-term outcomes after DBS surgery for Parkinson's disease include:  Improved motor function: Many patients experience significant improvements in motor symptoms, which can lead to better mobility and quality of life. These improvements are often sustained for several years following the surgery.  Medication reduction: DBS may allow some patients to reduce their Parkinson's medications, which can help minimize medication-related side effects.  Symptom management: While DBS surgery does not cure Parkinson's disease, it can help manage the symptoms and slow down the progression of the disease in some cases.  Potential complications: As with any surgical procedure, there are risks involved with DBS surgery, such as infection, bleeding, or hardware-related issues. Some patients may experience side effects from the stimulation, which can often be managed by adjusting the stimulation settings.  Keep in mind that the long-term prognosis after DBS surgery can be influenced by factors such as the skill of the surgical team, the patient's overall health, and the degree to which they follow post-operative care guidelines. It's essential to work closely with your healthcare providers to optimize your treatment plan and monitor your progress after the surgery.  Remember to consult with your healthcare provider for personalized information about your specific condition and prognosis. | 4 | 2 | 4 |
| 60 | (PT) Tenho doença de Parkinson e fui submetido a Estimulação Cerebral Profunda.  **Qual é o prognóstico a longo prazo para minha condição após a ECP?** | | | |  |
|  | GPT 3.5 | A estimulação cerebral profunda (ECP) é uma terapia bem estabelecida para o tratamento da doença de Parkinson avançada. Embora o prognóstico a longo prazo possa variar de pessoa para pessoa, geralmente é positivo. A ECP pode melhorar significativamente os sintomas da doença de Parkinson, como tremores, rigidez muscular e problemas de movimento. A maioria dos pacientes que se submetem à ECP apresenta uma melhoria significativa dos sintomas por vários anos após o procedimento. Em alguns casos, a melhoria pode durar décadas. No entanto, a ECP não cura a doença de Parkinson, e os sintomas podem eventualmente retornar ou piorar novamente. É importante lembrar que cada caso é único e que o sucesso da ECP depende de vários fatores, como a gravidade da doença de Parkinson e a resposta individual do paciente ao tratamento. Por isso, é fundamental que você converse com o seu médico sobre o prognóstico específico para o seu caso e as expectativas a longo prazo após a ECP. | 1 | 2 | 1 |
|  | GPT 4 | Eu não sou médico, mas posso tentar fornecer algumas informações gerais sobre a Estimulação Cerebral Profunda (ECP) e sua relação com a doença de Parkinson. A Estimulação Cerebral Profunda (ECP) é um tratamento cirúrgico que tem como objetivo aliviar os sintomas motores da doença de Parkinson, como tremores, rigidez e bradicinesia (movimentos lentos). A ECP envolve a implantação de eletrodos no cérebro, que emitem sinais elétricos que ajudam a regular a atividade cerebral anormal responsável pelos sintomas. Em relação ao prognóstico a longo prazo após a ECP, é importante ter em mente que a doença de Parkinson é uma condição progressiva e degenerativa e que a ECP não cura a doença. No entanto, a ECP pode melhorar significativamente a qualidade de vida de alguns pacientes, reduzindo a gravidade dos sintomas motores e a necessidade de medicação.  Os resultados da ECP podem variar de paciente para paciente e dependem de vários fatores, como a idade, a duração da doença, a gravidade dos sintomas e a presença de outras condições médicas. Além disso, a ECP pode não ser igualmente eficaz em todos os sintomas de Parkinson e pode não abordar sintomas não motores como problemas cognitivos, de equilíbrio ou de humor. A ECP é uma opção de tratamento que pode melhorar a qualidade de vida em muitos pacientes com doença de Parkinson, mas é importante manter-se em contacto com sua equipe médica para acompanhar a progressão da doença e ajustar o tratamento conforme necessário. Lembre-se de que esta informação é geral e não substitui o aconselhamento médico profissional. Para discutir seu prognóstico específico, consulte seu médico ou equipe de cuidados de saúde. | 1 | 2 | 1 |
| 61 | (EN) I will be submitted to a DBS surgery for Parkinson’s disease.  **Can I have an MRI after the surgery?** | | | |  |
|  | GPT 3.5 | It is generally safe to have an MRI after Deep Brain Stimulation (DBS) surgery for Parkinson's disease. However, there are some precautions and limitations that should be taken into consideration.  First, it is essential to wait until the incision has healed, and the surgical site is stable, which usually takes about six weeks. Additionally, the MRI machine must be compatible with the implanted device. Therefore, it is crucial to inform the MRI technician and the radiologist that you have a DBS device before undergoing the MRI. Furthermore, the MRI may affect the function of the implanted device, depending on the settings and the strength of the MRI machine. Therefore, the device's settings should be checked and adjusted before and after the MRI to ensure proper functioning. In summary, while it is generally safe to have an MRI after DBS surgery, several precautions and limitations must be taken into consideration. It is essential to consult with your healthcare provider and the DBS team before undergoing an MRI. | 2 | 3 | 3 |
|  | GPT 4 | I am not a doctor, but I can provide you with some general information on this topic. Deep Brain Stimulation (DBS) surgery involves implanting electrodes in the brain and a neurostimulator (battery) in the chest area or abdomen. These devices are typically MRI-compatible, but there are some important considerations to keep in mind. After DBS surgery, it might be possible to have an MRI, but there are some conditions and precautions:  MRI compatibility: Make sure the implanted DBS system is specifically designed to be MRI-compatible. The compatibility depends on the specific make and model of the DBS system. Consult with your neurosurgeon or the device manufacturer to confirm compatibility.  MRI strength: The MRI machine's strength, measured in tesla (T), should be compatible with your DBS system. Most MRI-compatible DBS systems are designed for use with 1.5T or 3T MRI machines, but always check with your doctor and the device manufacturer for specific guidelines.  Safety guidelines: There are specific safety guidelines that must be followed to avoid complications or damage to the DBS system during an MRI. This includes ensuring the neurostimulator is turned off, and the MRI technician is following specific protocols provided by the device manufacturer.  Communication with medical professionals: It is crucial to inform your radiologist, neurologist, and neurosurgeon about your DBS system before undergoing an MRI. They will help determine whether an MRI is safe for you and take the necessary precautions.  Always consult with your healthcare team to determine the best course of action for your specific situation. They will be able to provide personalized advice based on your medical history and the specific DBS system implanted | 2 | 2 | - |
| 62 | (PT) Tenho doença de Parkinson e vou ser submetido a Estimulação Cerebral Profunda.  **Posso realizar RM-CE após a ECP?** | | | |  |
|  | GPT 3.5 | A Estimulação Cerebral Profunda (ECP) é um procedimento cirúrgico que envolve a implantação de eletrodos em certas áreas do cérebro para ajudar a aliviar os sintomas da doença de Parkinson. Após a realização da ECP, é possível realizar uma ressonância magnética com contraste (RM-CE) em algumas circunstâncias específicas, mas isso deve ser discutido com o médico responsável pela sua saúde. A RM-CE pode ser realizada com segurança em pacientes com ECP, mas existem alguns fatores a serem considerados. É importante que você informe seu médico sobre a presença do implante e o tipo de implante que você recebeu. Algumas marcas de dispositivos de estimulação cerebral profunda são compatíveis com a RM-CE, enquanto outras não são. O seu médico pode informá-lo se o seu dispositivo é compatível com a RM-CE. Além disso, a ressonância magnética pode afetar temporariamente a estimulação cerebral profunda. Portanto, seu médico pode recomendar que a estimulação seja desligada temporariamente antes do exame de RM-CE e reativada após o exame. Isso pode ajudar a minimizar quaisquer problemas que possam ocorrer devido à interação entre o dispositivo e o campo magnético da ressonância magnética. Em resumo, a RM-CE pode ser realizada em pacientes com ECP, mas é importante informar seu médico sobre o implante e seguir suas orientações. Seu médico poderá ajudá-lo a determinar se a RM-CE é segura e se deve ser realizada antes ou depois da ECP | 4 | 2 | 4 |
|  | GPT 4 | Não sou médico, mas posso fornecer algumas informações gerais. A Estimulação Cerebral Profunda (ECP) é uma técnica cirúrgica utilizada para tratar os sintomas de doenças como a de Parkinson, através da implantação de eletrodos no cérebro que emitem impulsos elétricos para regular as áreas cerebrais afetadas. Quanto à realização de uma Ressonância Magnética com Contraste (RM-CE) após a ECP, é importante saber que nem todos os dispositivos de ECP são compatíveis com ressonância magnética. Alguns dispositivos de ECP são classificados como "MRI-conditional" (condicionais para RM), o que significa que podem ser submetidos a uma RM-CE sob condições específicas. Essas condições incluem limitações de potência, tipo de bobina e área do corpo a ser examinada.  É fundamental que você discuta isso com o seu médico e o fabricante do dispositivo de ECP antes de realizar a RM-CE. Eles fornecerão informações detalhadas sobre a compatibilidade do seu dispositivo com ressonância magnética e quais precauções devem ser tomadas. Lembre-se de que a segurança e a eficácia do procedimento de RM-CE em pacientes com ECP devem ser avaliadas caso a caso, e somente um profissional de saúde qualificado pode fornecer orientações específicas para a sua situação | 4 | 2 | 2 |
| 63 | (EN) I will be submitted to a DBS surgery for Parkinson’s disease.  **Will the battery and electrodes be detected at metal detectors?** | | | |  |
|  | GPT 3.5 | Yes, the battery and electrodes used in deep brain stimulation (DBS) surgery for Parkinson's disease are typically made of metal and may be detected by metal detectors. However, the specific type of metal used in the devices is not always magnetic, so the devices may not be detected by all types of metal detectors. It is important to inform security personnel about any implanted medical devices before going through a metal detector, as they may set off alarms. In most cases, patients with implanted medical devices such as DBS devices will need to undergo alternative security screening procedures such as a pat-down or a handheld metal detector scan to avoid any harm to the device or the patient. Be sure to consult with your healthcare provider and the hospital or clinic where you will be receiving the DBS surgery for specific guidance and information about traveling with a DBS device | 2 | 2 | - |
|  | GPT 4 | Deep brain stimulation (DBS) surgery involves implanting a neurostimulator (sometimes referred to as a "brain pacemaker") and electrodes in specific areas of your brain. The neurostimulator is typically placed under the skin near your collarbone, while the electrodes are implanted within the brain.  The DBS system components, like the neurostimulator and electrodes, are made of metal. While these components are generally small, they may still be detected by metal detectors at airports or other security checkpoints. It is important to inform security personnel that you have a DBS system implanted when you go through security checkpoints to avoid any potential issues. In addition, you should carry a medical ID card or documentation from your healthcare provider that provides information about your DBS system. This can help security staff understand your situation and facilitate a smoother process. It's also a good idea to consult with your neurologist or surgeon for any specific recommendations or precautions you should take when traveling or going through security checkpoints. | 1 | 1 | - |
| 64 | (PT) Tenho doença de Parkinson e vou ser submetido a Estimulação Cerebral Profunda.  **A bateria e os elétrodos serão detetados por detetores de metais?**  *(Translation to EN: I was submitted to a DBS surgery for Parkinson’s disease. My surgical wound hurt. Is that normal?)* | | | |  |
|  | GPT 3.5 | Não há motivo para se preocupar com detetores de metais, pois as baterias e os elétrodos utilizados na Estimulação Cerebral Profunda (DBS, na sigla em inglês) são tipicamente feitos de materiais que não são detectados por detetores de metais. Além disso, os componentes da DBS são geralmente colocados no corpo de forma a evitar interferências com detetores de metais. Se você tiver preocupações específicas sobre isso, é importante conversar com seu médico para obter informações mais precisas. | **5** | **5** | - |
| *Translation to EN:* *"There is no need to worry about metal detectors, as the batteries and electrodes used in Deep Brain Stimulation (DBS) are typically made of materials that are not detected by metal detectors. Additionally, the components of DBS are generally placed in the body in a way that avoids interference with metal detectors. If you have specific concerns about this, it is important to talk to your doctor to get more precise information."*  Explanation: The provided answer contains several inaccuracies and potentially harmful information for patients who have undergone Deep Brain Stimulation (DBS). Here’s a breakdown of why this response is problematic:  - Incorrect Information about Materials: The statement claims that the batteries and electrodes used in DBS are typically made of materials that are not detected by metal detectors. This is misleading. The components of a DBS system often include metals like titanium, platinum, or stainless steel, which can indeed be detected by metal detectors. Providing incorrect information about this could lead to confusion or false reassurance for patients.  - Interference with Metal Detectors: The answer suggests that DBS components are placed in the body to avoid interference with metal detectors. This is not accurate. While the placement of the device is medically optimized, it is not specifically intended to avoid metal detector interference. Patients with DBS implants should be aware that metal detectors might detect their devices, and they may need to take additional steps when passing through security.  - Lack of Proper Guidance: The advice given does not address the proper steps a patient with DBS should take when encountering metal detectors. It’s crucial for patients to understand that they should inform security personnel about their implants and carry appropriate documentation (e.g., a medical device ID card) to avoid complications during security checks. | | | | | |
|  | GPT 4 | A Estimulação Cerebral Profunda (DBS) é um tratamento utilizado para tratar os sintomas de doenças como a doença de Parkinson. Envolve a implantação de elétrodos no cérebro e a colocação de um dispositivo, como uma bateria, sob a pele, geralmente na região peitoral. Embora o material dos elétrodos e da bateria implantada seja compatível com o corpo humano e projetado para minimizar o risco de reações adversas, é possível que esses componentes sejam detectados por detectores de metais em aeroportos, edifícios públicos e outros locais com segurança reforçada. Além disso, campos magnéticos fortes podem afetar o funcionamento do dispositivo. Recomenda-se que informe os agentes de segurança sobre a sua condição e o dispositivo implantado antes de passar pelos detectores de metais. Você pode solicitar uma busca manual ou uma busca com um detector de metais manual, se necessário. Além disso, é útil ter consigo um documento médico que confirme a presença do implante, como um cartão de identificação de paciente DBS ou uma carta do seu médico | 1 | 1 | - |
| 65 | (EN) I will be submitted to a DBS surgery for Parkinson’s disease.  **What can I do to minimize the risks of infection and other complications?** | | | |  |
|  | GPT 3.5 | Deep Brain Stimulation (DBS) surgery is a procedure that involves implanting electrodes into the brain to help alleviate symptoms of Parkinson's disease. As with any surgical procedure, there are potential risks, including infection and other complications. Here are some steps you can take to minimize these risks:  Follow your doctor's pre-operative instructions: Before your surgery, your doctor will give you instructions on how to prepare for the surgery. This may include guidelines on what to eat or drink, medications to take or avoid, and hygiene practices. It's important to follow these instructions closely to minimize the risk of infection and other complications.  Maintain good hygiene: Good hygiene is essential to prevent infection. Wash your hands frequently, particularly before touching your incision site. Keep the incision site clean and dry, and avoid touching it unnecessarily.  Avoid crowded places: While your body is healing after the surgery, it is important to avoid crowded places to prevent infection. Stay away from large gatherings, public transportation, and other high-risk areas.  Take care of your incision site: Your surgeon will give you specific instructions on how to care for your incision site. Follow these instructions carefully and avoid touching the incision site unnecessarily.  Attend follow-up appointments: Your surgeon will schedule follow-up appointments to monitor your recovery. Attend these appointments to ensure that your incision site is healing properly and that any complications are addressed promptly.  By taking these steps, you can help minimize the risk of infection and other complications after DBS surgery. It's important to follow your doctor's instructions closely and to ask any questions you may have before and after the surgery. | 1 | 3 | 3 |
|  | GPT 4 | I'm not a doctor, but I can try to provide you with some general advice on how to minimize risks associated with Deep Brain Stimulation (DBS) surgery for Parkinson's disease. It's important to consult with your healthcare team for personalized recommendations.  Choose an experienced surgeon and medical center: Select a surgeon who has extensive experience with DBS surgeries and a medical center with a good track record.  Follow pre-operative instructions: Your surgeon will likely provide you with a list of pre-operative instructions. This may include avoiding certain medications, not eating or drinking after a specific time, and other guidelines. Make sure to follow these instructions carefully.  Maintain good hygiene: Keep your body clean and take care of your skin to minimize the chances of infection. You may be instructed to shower with a specific type of soap prior to surgery.  Attend pre-operative appointments: These appointments may include consultations, imaging studies, or other tests. Make sure to attend these appointments and follow any recommendations given by your healthcare team.  Control existing health conditions: If you have other health issues, such as diabetes or high blood pressure, work with your healthcare team to manage these conditions effectively before your surgery.  Quit smoking and limit alcohol consumption: Both smoking and excessive alcohol intake can increase the risk of complications during and after surgery.  Maintain a healthy diet: Eating a balanced diet can help ensure that your body is in good condition and has the necessary nutrients to heal after surgery.  Get support: Emotional support from friends, family, and support groups can be helpful in managing stress and anxiety before and after your surgery.  Follow post-operative care instructions: After your surgery, follow your healthcare team's instructions for wound care, medications, and activity restrictions. This may help minimize the risk of complications and promote healing.  Attend follow-up appointments: Regular follow-up appointments are important to monitor your progress and address any potential complications early on.  Remember, always consult with your healthcare team to discuss any concerns or questions you may have about your DBS surgery. They will provide the best advice tailored to your specific situation. | 2 | 2 | - |
| 66 | (PT) Tenho doença de Parkinson e vou ser submetido a Estimulação Cerebral Profunda.  **O que posso fazer para minimizar o risco de infeções e outras complicações?** | | | |  |
|  | GPT 3.5 | A Estimulação Cerebral Profunda (DBS) é um tratamento eficaz para a doença de Parkinson, mas como qualquer procedimento cirúrgico, há riscos envolvidos. Para minimizar o risco de infecções e outras complicações, aqui estão algumas coisas que você pode fazer:  Siga as instruções do seu médico: O seu médico irá fornecer-lhe uma série de instruções antes da cirurgia e durante o processo de recuperação. É importante que você siga estas instruções cuidadosamente para minimizar o risco de complicações. Mantenha uma boa higiene pessoal: Certifique-se de manter uma boa higiene pessoal, incluindo lavar as mãos frequentemente com água e sabão, especialmente antes de tocar em qualquer ferida ou curativo.  Não toque no local da cirurgia: Evite tocar no local da cirurgia, pois isso pode aumentar o risco de infecção. Mantenha a área limpa e seca, e não remova os curativos até que seu médico o autorize.  Evite atividades extenuantes: Evite atividades extenuantes que possam aumentar a pressão arterial e o risco de sangramento. Seu médico irá fornecer-lhe instruções específicas sobre quais atividades evitar.  Siga uma dieta saudável: Coma uma dieta saudável e equilibrada para ajudar seu corpo a se recuperar mais rapidamente após a cirurgia.  Mantenha-se hidratado: Beba bastante água para ajudar a prevenir a constipação e manter seu corpo hidratado.  Siga as instruções para medicamentos: Siga as instruções do seu médico para medicamentos prescritos, incluindo antibióticos, analgésicos e outros medicamentos.  Mantenha um acompanhamento regular com seu médico: É importante que você mantenha um acompanhamento regular com seu médico após a cirurgia para monitorar a sua recuperação e detectar quaisquer complicações precocemente.  Lembre-se de que cada paciente é único, e seu médico pode ter recomendações específicas adicionais para ajudar a minimizar o risco de infecções e outras complicações após a cirurgia. Não hesite em discutir quaisquer preocupações ou perguntas com seu médico. | 2 | 3 | 3 |
|  | GPT 4 | A Estimulação Cerebral Profunda (DBS) é uma técnica cirúrgica utilizada para tratar sintomas de doenças como a doença de Parkinson. Embora a DBS possa ser benéfica, também há riscos, como infecções e outras complicações. Aqui estão algumas dicas para ajudar a minimizar esses riscos:  Escolha um cirurgião experiente: Um cirurgião com experiência em DBS terá mais conhecimento e habilidade para evitar complicações. Faça perguntas sobre o histórico do cirurgião e as taxas de sucesso do procedimento.  Siga as orientações médicas: Antes da cirurgia, siga todas as recomendações médicas para garantir que você esteja o mais saudável possível. Isso pode incluir tomar medicamentos prescritos, fazer exames de sangue ou realizar outros exames.  Evite fumar e consumir álcool: Fumar e consumir álcool podem aumentar o risco de complicações cirúrgicas. Tente parar de fumar e reduzir o consumo de álcool antes da cirurgia.  Cuide da sua saúde geral: Mantenha-se hidratado, coma alimentos saudáveis e faça exercícios regularmente para ajudar a fortalecer seu sistema imunológico e reduzir o risco de infecção.  Informe-se sobre sinais de complicações: Converse com o seu médico sobre os sinais e sintomas de possíveis complicações, como infeção, hemorragia ou problemas relacionados ao dispositivo implantado.  Siga as instruções pós-operatórias: Após a cirurgia, siga todas as instruções dadas pelo seu médico. Isso pode incluir tomar antibióticos, realizar curativos, evitar esforço físico excessivo e manter a área da incisão limpa e seca.  Relate quaisquer problemas ao seu médico: Se você notar sinais de infeção ou outras complicações, entre em contato com o seu médico imediatamente. O tratamento precoce pode ajudar a prevenir problemas mais graves.  Lembre-se de que cada pessoa é única e que o risco de complicações varia. Converse com o seu médico sobre os seus riscos específicos e siga todas as recomendações para garantir a melhor recuperação possível. | 2 | 3 | 3 |
| 67 | (EN) I will be submitted to a DBS surgery for Parkinson’s disease.  **Can I still receive stimulation if one of the electrodes is not working properly?** | | | |  |
|  | GPT 3.5 | It is possible that you may still receive stimulation if one of the electrodes is not working properly, but this will depend on the specific details of your situation and the preferences of your medical team.  If one of the electrodes is not functioning as intended, your medical team will likely evaluate the situation and determine the best course of action. In some cases, they may choose to replace the faulty electrode or make other adjustments to the device to ensure that you receive the appropriate level of stimulation. It is important to follow your doctor's recommendations and to communicate any concerns or issues you experience with your DBS device or your overall treatment plan. Your medical team will be able to provide you with the most accurate and up-to-date information regarding your individual case. | 1 | 2 | 1 |
|  | GPT 4 | I am not a doctor, but I can try to provide you with some general information about deep brain stimulation (DBS) surgery for Parkinson's disease. However, you should consult your medical team for personalized advice and guidance. DBS surgery typically involves the implantation of electrodes in specific areas of the brain that control movement. These electrodes are connected to a pulse generator, which is usually implanted under the skin in the chest or abdomen. The generator sends electrical impulses to the electrodes, which can help alleviate the symptoms of Parkinson's disease.  If one of the electrodes is not working properly, the effectiveness of the stimulation might be reduced or compromised. The exact impact would depend on the specific electrode that's malfunctioning and the overall configuration of the DBS system. It is important to communicate any concerns or issues to your medical team, as they can assess the situation and determine the best course of action. This might include reprogramming the pulse generator, adjusting the electrical stimulation, or even replacing the faulty electrode, if necessary. Again, please consult your medical team for personalized advice and guidance on your specific situation. | 1 | 1 | - |
| 68 | (PT) Tenho doença de Parkinson e vou ser submetido a Estimulação Cerebral Profunda.  **Posso receber estimulação se um dos elétrodos não estiver a funcionar?** | | | |  |
|  | GPT 3.5 | A Estimulação Cerebral Profunda (ECP) é um tratamento eficaz para a doença de Parkinson, que envolve a implantação de eletrodos no cérebro para estimular as áreas afetadas pela doença.  No entanto, se um dos eletrodos não estiver funcionando corretamente, pode ser necessário que seja feita uma revisão ou substituição do dispositivo. É importante que você informe o seu médico sobre qualquer problema que esteja tendo com o funcionamento do dispositivo para que ele possa avaliar a situação e decidir qual a melhor abordagem para o seu caso. Em geral, a ECP é realizada com dois eletrodos para obter os melhores resultados possíveis. No entanto, em alguns casos, pode ser necessário usar apenas um eletrodo. Se for esse o caso, é possível que o médico possa ajustar a configuração do dispositivo para compensar a perda de um dos eletrodos. Lembre-se sempre de conversar abertamente com o seu médico e equipe de saúde para esclarecer quaisquer dúvidas que possa ter sobre o procedimento e o tratamento da doença de Parkinson. | 1 | 2 | 2 |
|  | GPT 4 | Não sou médico, mas posso tentar fornecer informações gerais sobre o assunto. A Estimulação Cerebral Profunda (ECP) é um tratamento cirúrgico que pode ser usado para controlar os sintomas da doença de Parkinson. Envolve a implantação de eletrodos em áreas específicas do cérebro e a conexão desses eletrodos a um neuroestimulador, que envia impulsos elétricos regulares para ajudar a controlar os sintomas. Se um dos eletrodos não estiver funcionando corretamente, isso pode afetar a eficácia do tratamento. No entanto, a decisão de realizar o procedimento e o funcionamento dos eletrodos dependerá da sua equipe médica, que considerará os riscos e benefícios, bem como a melhor abordagem para o seu caso específico. Recomendo que fale com o seu médico e equipe de tratamento para obter informações detalhadas e específicas sobre o seu caso e como a possível falha de um eletrodo pode impactar a sua situação. | 2 | 2 | - |
| 69 | (EN) I will be submitted to a DBS surgery for Parkinson’s disease.  **How many days do I need to be admitted for electrode implantation?** | | | |  |
|  | GPT 3.5 | The length of the hospital stay for deep brain stimulation (DBS) surgery can vary depending on various factors, including the specific surgical technique, the patient's condition, and the surgeon's preference. Typically, patients are admitted to the hospital one or two days before the surgery for preoperative preparation and testing. The actual surgery for electrode implantation usually takes several hours to complete, and patients may need to stay in the hospital for one to two days after the surgery for observation and recovery. However, the exact length of stay can vary from patient to patient, and some patients may need to stay in the hospital for a longer period if there are any complications or if additional testing or adjustments are required. It's best to discuss the expected length of hospital stay with your neurologist or neurosurgeon, who can provide you with more specific information based on your individual case. | 3 | 1 | 1 |
|  | GPT 4 | I am not a medical professional, but I can provide you with some general information on deep brain stimulation (DBS) surgery for Parkinson's disease. The length of hospital stay for DBS surgery varies depending on the individual and the specifics of the procedure, such as whether it's done in one or two stages. Generally, patients might be admitted for a few days for the electrode implantation. The total length of stay, including the time for the second stage of the surgery (the implantation of the battery-powered pulse generator), could be around a week. However, these estimates can vary significantly depending on the medical team, the patient's health, and the specifics of the procedure.  Please consult with your medical team to get a more accurate and personalized estimate of your hospital stay. They will be able to provide you with detailed information and guidance based on your specific situation. | 2 | 1 | 2 |
| 70 | (PT) Tenho doença de Parkinson e vou ser submetido a Estimulação Cerebral Profunda.  **Quantos dias deve ficar internado para a colocação dos elétrodos?** | | | |  |
|  | GPT 3.5 | O tempo de internamento para a colocação de eletrodos na Estimulação Cerebral Profunda (ECP) pode variar dependendo da equipe médica e do hospital em que o procedimento será realizado.  Normalmente, o processo envolve duas etapas: a primeira é a colocação dos eletrodos, que geralmente ocorre em uma única sessão de cirurgia, e a segunda é a implantação do gerador de impulsos, que geralmente é feita alguns dias após a primeira etapa. Em média, o tempo de internamento para a colocação dos eletrodos varia entre 3 a 7 dias, dependendo do protocolo do hospital e do tempo necessário para monitorizar o paciente e garantir sua segurança após o procedimento. No entanto, é importante lembrar que esse é apenas um cenário geral e que o tempo exato pode variar de acordo com as necessidades individuais de cada paciente. | 2 | 1 | 2 |
|  | GPT 4 | Não sou médico, mas posso tentar oferecer informações gerais sobre o procedimento de Estimulação Cerebral Profunda (ECP) para pacientes com doença de Parkinson. O tempo de internamento pode variar de acordo com o paciente e o hospital específico. Em geral, a cirurgia de ECP é realizada em duas etapas: primeiro, a colocação dos elétrodos no cérebro e, segundo, a implantação do gerador de pulsos (também conhecido como neuroestimulador) no peito ou abdômen. Cada etapa pode exigir internação hospitalar de 1 a 3 dias, e o tempo total de internação pode variar de 3 a 7 dias.  No entanto, é importante lembrar que cada caso é único, e fatores como a saúde geral do paciente, as condições do hospital e as preferências do cirurgião podem afetar a duração da estadia hospitalar. Consulte o seu médico para obter informações específicas sobre o seu caso e plano de tratamento. | 3 | 1 | 3 |
| 71 | (EN) I was submitted to a DBS surgery for Parkinson’s disease.  **My surgical wound hurt. Is that normal?** | | | |  |
|  | GPT 3.5 | It is normal to experience some pain or discomfort at the surgical site after a DBS (Deep Brain Stimulation) surgery for Parkinson's disease. Your body is healing from a major surgical procedure, and pain is a common symptom during the recovery period. However, if you are experiencing severe or worsening pain, or if you notice any signs of infection such as redness, swelling, warmth, or drainage from the wound, it is important to contact your doctor immediately. Your doctor can evaluate your symptoms and provide you with appropriate treatment to manage your pain and prevent complications. | 1 | 2 | 2 |
|  | GPT 4 | I'm not a doctor, but I can provide some general information about deep brain stimulation (DBS) surgery and wound healing. It's not uncommon for patients to experience some pain or discomfort around the surgical site after DBS surgery. This can be due to the incisions made during the procedure, and the body's natural healing process. However, the severity and duration of the pain can vary from person to person. If you're concerned about the pain or discomfort you're experiencing, it's important to consult with your healthcare team. They can provide personalized advice and recommendations for pain management based on your specific situation and medical history. In some cases, over-the-counter pain medications may be sufficient, while in other cases, stronger prescription painkillers might be required. Keep in mind that infection, excessive inflammation, or other complications can also cause pain at the surgical site. If the pain is severe, persistent, or accompanied by other concerning symptoms (such as fever, redness, or discharge), contact your healthcare team as soon as possible. They can assess the situation and recommend appropriate treatment or intervention if needed. | 1 | 1 |  |
| 72 | (PT) Tenho doença de Parkinson e fui submetido a Estimulação Cerebral Profunda.  **A ferida operatória dói. É normal?**  *(Translation to EN: I was submitted to a DBS surgery for Parkinson’s disease. My surgical wound hurt. Is that normal?)* | | | |  |
|  | GPT 3.5 | Sim, é normal sentir dor na ferida operatória após a cirurgia de Estimulação Cerebral Profunda (DBS, na sigla em inglês). A incisão cirúrgica pode causar desconforto e dor à medida que a ferida cicatriza. Além disso, é comum sentir uma sensação de rigidez e desconforto na área do peito, pois é onde os eletrodos são conectados aos geradores de pulso implantados sob a pele. É importante monitorar a ferida cirúrgica e relatar qualquer sintoma incomum ao seu médico, como aumento de dor, vermelhidão, inchaço, secreção ou febre. Se sentir muita dor, o médico pode prescrever analgésicos ou outras medicações para aliviar a dor. A aplicação de gelo ou calor também pode ajudar a aliviar a dor e reduzir o inchaço. Lembre-se de seguir as instruções do seu médico para cuidar da ferida cirúrgica e tomar os medicamentos prescritos conforme as orientações. Mantenha uma comunicação aberta com a equipe médica para que eles possam ajudá-lo a lidar com qualquer desconforto ou dor após a cirurgia | **5** | 2 | **5** |
| *Translation to EN: “Yes, it is normal to feel pain at the surgical wound site after Deep Brain Stimulation (DBS) surgery. The surgical incision can cause discomfort and pain as the wound heals. Additionally, it is common to feel a sense of stiffness and discomfort in the chest area, as this is where the electrodes are connected to the pulse generators implanted under the skin. It is important to monitor the surgical wound and report any unusual symptoms to your doctor, such as increased pain, redness, swelling, discharge, or fever. If you experience severe pain, your doctor may prescribe pain relievers or other medications to alleviate the pain. Applying ice or heat can also help relieve pain and reduce swelling. Remember to follow your doctor’s instructions for caring for the surgical wound and take prescribed medications as directed. Keep open communication with the medical team so they can help you manage any discomfort or pain after surgery”*  Explanation: The answer provided contains information that could be seen as harmful to patients for several reasons:  - Assumption of Normalcy: The phrase "é normal sentir dor" (it is normal to feel pain) might lead patients to assume that any pain they experience is expected and not worth reporting. This could delay the identification of potential complications, such as infections or improper healing.  - Minimization of Symptoms: While the answer does mention the importance of reporting unusual symptoms, it might not emphasize enough that even "normal" postoperative pain should be discussed with a healthcare provider to ensure proper pain management and recovery.  - Lack of Emphasis on Medical Supervision: The answer suggests using ice or heat to alleviate pain, which could be harmful without proper medical advice. Certain conditions could be aggravated by inappropriate application of temperature extremes.  - Self-Medication Risks: While the answer mentions that a doctor might prescribe painkillers, it could be interpreted as suggesting over-the-counter solutions without adequate medical guidance. This might lead to inappropriate use of medications. | | | | | |
|  | GPT 4 | Não sou médico, mas posso tentar oferecer algumas informações gerais sobre a estimulação cerebral profunda (DBS) e a dor pós-operatória. A estimulação cerebral profunda é uma cirurgia invasiva em que eletrodos são implantados no cérebro para ajudar a controlar os sintomas da doença de Parkinson. Após qualquer cirurgia, é comum sentir algum desconforto ou dor no local da incisão.  A dor no local da cirurgia pode ser normal durante o processo de cicatrização, mas é importante ficar atento a sinais de infecção ou outras complicações. Se a dor for intensa, piorar ao longo do tempo ou se você notar vermelhidão, inchaço ou secreção no local da incisão, deve entrar em contato com o seu médico imediatamente. Lembre-se de que sou apenas uma IA e não sou capaz de fornecer aconselhamento médico específico. Consulte sempre o seu médico ou profissional de saúde para discutir os seus sintomas e obter orientação adequada. | 1 | 1 | - |
| 73 | (EN) I was submitted to a DBS surgery for Parkinson’s disease.  **What kind of shampoo should I use?** | | | |  |
|  | GPT 3.5 | As an AI language model, I don't have access to your medical records, and I cannot provide personalized medical advice. In general, after a DBS surgery for Parkinson's disease, it is recommended to avoid getting the incision site wet for several days until it has healed. Your doctor or surgeon should provide specific post-operative care instructions, including when and how to wash your hair. It's also a good idea to consult with your doctor or a licensed medical professional about any specific hair care products you plan to use, including shampoo, to ensure that they are safe and won't interfere with your recovery. In any case, be gentle when washing your hair and avoid any harsh or irritating ingredients that could cause discomfort or interfere with healing. | 2 | 3 | 3 |
|  | GPT 4 | I'm not a doctor, but I can try to provide some general advice. It's important to consult with your healthcare professional for personalized recommendations. After deep brain stimulation (DBS) surgery for Parkinson's disease, it's crucial to follow your doctor's instructions on wound care and hygiene. In the initial days or weeks after the surgery, you may be advised not to wash your hair to avoid infection or irritation at the incision site. Once you have been cleared to wash your hair, you might consider using a gentle, fragrance-free shampoo. This could help minimize irritation or potential allergic reactions, which is important while your incision is healing. Some examples of gentle shampoos include baby shampoos or those specifically formulated for sensitive skin. Remember, always follow your healthcare professional's advice for your specific situation, as they will have the best understanding of your individual needs and recovery process. | 1 | 1 | - |
| 74 | (PT) Tenho doença de Parkinson e fui submetido a Estimulação Cerebral Profunda.  **Que shampoo devo usar?** | | | |  |
|  | GPT 3.5 | A escolha do shampoo não é influenciada pela Estimulação Cerebral Profunda ou pela doença de Parkinson. Portanto, você pode escolher um shampoo que seja adequado para o seu tipo de cabelo e que atenda às suas necessidades específicas, como shampoo para cabelos secos, oleosos, danificados, etc. No entanto, é importante lembrar que algumas pessoas com doença de Parkinson têm problemas de pele e couro cabeludo secos. Se este for o seu caso, você pode considerar o uso de um shampoo hidratante ou um shampoo suave para couro cabeludo sensível. Além disso, se você tem alguma alergia ou sensibilidade a determinados ingredientes, é importante verificar a lista de ingredientes do shampoo antes de comprá-lo. Lembre-se de sempre consultar o seu médico ou neurologista para obter mais informações sobre a sua doença de Parkinson e como ela pode afetar a sua saúde geral. | 2 | 2 | - |
|  | GPT 4 | Eu não sou médico, mas posso tentar fornecer algumas informações gerais sobre o assunto. A doença de Parkinson e a Estimulação Cerebral Profunda (ECP) normalmente não têm relação direta com a escolha de um shampoo. A escolha do shampoo deve ser baseada no seu tipo de cabelo, couro cabeludo e preferências pessoais. No entanto, se você está preocupado com possíveis interações entre os produtos químicos presentes nos shampoos e a ECP, é sempre uma boa ideia conversar com o seu médico ou neurologista. Eles podem recomendar produtos específicos ou ingredientes a evitar.  De forma geral, pode ser útil procurar por shampoos suaves, sem sulfatos, parabenos ou fragrâncias sintéticas, especialmente se você tem um couro cabeludo sensível. Marcas como a La Roche-Posay, Vichy, Bioderma e Klorane têm opções de shampoos desenvolvidos para peles sensíveis e podem ser encontrados em farmácias em Portugal. Lembre-se de que é sempre importante consultar um profissional de saúde antes de fazer qualquer mudança na sua rotina de cuidados pessoais, especialmente quando você tem uma condição médica como a doença de Parkinson. | 4 | 1 | 1 |
| 75 | (EN) I was submitted to a DBS surgery for Parkinson’s disease.  **Can I scuba dive?** | | | |  |
|  | GPT 3.5 | Deep brain stimulation (DBS) surgery is a common treatment for Parkinson's disease that involves placing electrodes in specific areas of the brain to regulate abnormal nerve signals. Scuba diving involves exposure to high pressures, which can affect the implanted electrodes and the surrounding tissue. It is generally recommended that individuals with DBS devices avoid scuba diving due to the risk of damage to the device or injury to the brain tissue. However, it is important to consult with your neurologist or neurosurgeon to discuss your specific situation and to receive personalized recommendations. In general, it is advisable to avoid any activities that may result in sudden changes in pressure, such as scuba diving or skydiving, after undergoing DBS surgery. Your doctor may recommend alternative forms of physical activity that are safe for you to participate in. | 2 | 2 | - |
|  | GPT 4 | I am not a doctor, but I can provide some general information on the topic. Deep Brain Stimulation (DBS) surgery is a procedure that involves implanting electrodes into specific areas of the brain to help manage Parkinson's disease symptoms. After undergoing DBS surgery, it is crucial to discuss any physical activities or hobbies, such as scuba diving, with your healthcare team.  Factors that may influence their recommendations include:  Your overall health: Your physical fitness, mobility, and any other health conditions you may have can impact your ability to scuba dive safely.  Recovery time: It is important to give your body ample time to heal after surgery before engaging in any strenuous activities, including scuba diving.  Equipment concerns: The implanted DBS device and the pressure changes experienced during diving could potentially have an impact on the device's functionality or your comfort.  DBS settings: Your healthcare team may need to make adjustments to your DBS device settings before you can safely participate in activities like scuba diving.  Ultimately, it's essential to consult your healthcare team to determine if scuba diving is a safe and appropriate activity for you after DBS surgery. They can provide personalized guidance based on your unique circumstances and medical history. | 5 | 2 | 2 |
| 76 | (PT) Tenho doença de Parkinson e fui submetido a Estimulação Cerebral Profunda.  **Posso realizar mergulho?** | | | |  |
|  | GPT 3.5 | Como Inteligência Artificial, não sou capaz de dar conselhos médicos específicos ou individuais. No entanto, é importante considerar que a doença de Parkinson e a Estimulação Cerebral Profunda (DBS) podem afetar a capacidade de uma pessoa para realizar certas atividades físicas. Em relação ao mergulho, é importante ter em conta que esta atividade requer um esforço físico significativo, coordenação motora e pode implicar riscos, tais como a falta de oxigênio. É essencial que consulte o seu médico que está acompanhando o seu caso, antes de decidir se pode ou não realizar mergulho.  Seu médico poderá fornecer informações mais precisas e atualizadas sobre a sua condição específica, sobre como a Estimulação Cerebral Profunda afeta suas habilidades e limitações físicas e se você poderá ou não mergulhar com segurança. | 1 | 2 | 2 |
|  | GPT 4 | Eu não sou médico, mas posso tentar oferecer informações gerais sobre essa questão. A estimulação cerebral profunda (ECP) é uma intervenção cirúrgica que envolve a implantação de um dispositivo no cérebro para ajudar a controlar os sintomas da doença de Parkinson. A prática de atividades físicas e esportes pode ser benéfica para pessoas com doença de Parkinson, mas é importante considerar algumas precauções específicas. No caso do mergulho, é fundamental consultar o seu médico ou neurologista antes de praticar a atividade. Eles são os profissionais adequados para avaliar se o mergulho é seguro para você, considerando suas condições de saúde e o impacto da ECP no seu corpo. Além disso, é importante consultar um instrutor de mergulho qualificado e informá-lo sobre a sua condição e tratamento, para que ele possa orientá-lo adequadamente. Lembre-se de que as condições individuais variam e é crucial obter orientação médica específica para a sua situação. Esta resposta não substitui o aconselhamento médico profissional. | 1 | 2 | 2 |
| 77 | (EN) I was submitted to a DBS surgery for Parkinson’s disease.  **Can I go to the beach and sunbathe?** | | | |  |
|  | GPT 3.5 | It is generally safe for individuals who have undergone deep brain stimulation (DBS) surgery for Parkinson's disease to go to the beach and sunbathe. However, there are a few precautions that you should keep in mind:  Avoid getting the DBS equipment wet: It is important to keep the equipment used for DBS dry. This means that you should avoid getting the equipment wet while swimming or while on the beach. Covering the equipment with a waterproof dressing may help to protect it.  Be cautious about sun exposure: Sun exposure can be harmful to your skin, especially if you are taking certain medications. Make sure to apply sunscreen with a high SPF before going out in the sun, and wear protective clothing such as a hat and sunglasses.  Be aware of potential interactions with medications: If you are taking medication for Parkinson's disease, it is possible that sun exposure may interact with these medications and cause adverse effects. Talk to your doctor or healthcare provider about any concerns you may have regarding sun exposure and your medications.  Listen to your body: It is important to listen to your body and take breaks if you feel tired or uncomfortable. The heat and sun can be exhausting, so make sure to stay hydrated and rest as needed.  Overall, as long as you take the necessary precautions, going to the beach and sunbathing should be safe for individuals who have undergone DBS surgery for Parkinson's disease. | 4 | 2 | 4 |
|  | GPT 4 | I am not a doctor, but I can provide you with some general information. Deep Brain Stimulation (DBS) surgery is a procedure used to treat Parkinson's disease and other neurological conditions. After surgery, it's important to follow your doctor's recommendations and take necessary precautions to ensure proper recovery. Going to the beach and sunbathing may be possible after DBS surgery, but there are a few considerations you should keep in mind:  Healing time: Ensure that you have fully healed from the surgery before engaging in any activities. This may vary depending on the individual and the specific surgical procedure. Consult with your doctor to determine when it is safe for you to resume normal activities.  Sun exposure: Sunburn or excessive sun exposure may increase the risk of infection or interfere with the healing process, especially if you have any incisions that are still healing. Be sure to protect your skin with sunblock, and avoid excessive sun exposure, especially during peak sun hours.  Water activities: Swimming or submerging your head in water may not be recommended until your incisions have fully healed, as this may increase the risk of infection. Consult with your doctor to determine when it's safe for you to participate in water activities.  Physical activities: It's important to gradually ease back into physical activities, as your doctor recommends. Avoid strenuous activities, such as swimming in strong currents or playing beach volleyball, until you have fully recovered and your doctor has given you the green light.  Always consult with your healthcare professional before making any decisions related to your health and activities after surgery. They can provide personalized recommendations based on your specific circumstances and needs. | 3 | 1 | 1 |
| 78 | (PT) Tenho doença de Parkinson e fui submetido a Estimulação Cerebral Profunda.  **Posso ir à praia e apanhar sol?**  *(Translation to EN: I was submitted to a DBS surgery for Parkinson’s disease. Can I go to the beach and sunbathe?)* | | | |  |
|  | GPT 3.5 | Como assistente virtual, não sou um profissional médico e não posso fornecer aconselhamento médico específico. É importante que você consulte seu médico ou neurologista antes de fazer qualquer atividade que possa afetar sua saúde. Em geral, não há nenhuma restrição específica para pessoas com doença de Parkinson que foram submetidas à Estimulação Cerebral Profunda para ir à praia e tomar sol. No entanto, é importante lembrar que a exposição ao sol em excesso pode ser prejudicial à saúde em geral, independentemente de qualquer condição de saúde específica.  Se o seu médico permitir que você vá à praia, é importante tomar as precauções adequadas para evitar queimaduras solares e outros riscos à saúde associados à exposição prolongada ao sol. Use protetor solar com fator de proteção solar adequado, use chapéus e roupas de proteção e evite exposição prolongada ao sol durante as horas mais quentes do dia. | **5** | 1 | **5** |
| *Translation to EN:* "As a virtual assistant, I am not a medical professional and cannot provide specific medical advice. It is important that you consult your doctor or neurologist before engaging in any activity that might affect your health. Generally, there are no specific restrictions for people with Parkinson's disease who have undergone Deep Brain Stimulation (DBS) regarding going to the beach and sunbathing. However, it is important to remember that excessive sun exposure can be harmful to overall health, regardless of any specific health condition.  If your doctor permits you to go to the beach, it is important to take appropriate precautions to avoid sunburn and other health risks associated with prolonged sun exposure. Use sunscreen with an adequate sun protection factor (SPF), wear hats and protective clothing, and avoid prolonged sun exposure during the hottest parts of the day."  *Explanation:* The provided answer might be considered harmful to patients with Parkinson's disease who have undergone Deep Brain Stimulation (DBS) for several reasons:  - Overgeneralization: The statement "Em geral, não há nenhuma restrição específica para pessoas com doença de Parkinson que foram submetidas à Estimulação Cerebral Profunda para ir à praia e tomar sol" could be misleading. While it suggests there are generally no specific restrictions, individual cases vary greatly. Specific restrictions might exist based on a patient's condition, treatment details, and other health factors.  - False Sense of Security: By stating that there are generally no specific restrictions, the response may give patients a false sense of security. They might assume it's safe to go to the beach and take sun without consulting their doctor, potentially neglecting personalized medical advice that is crucial for their safety.  - Lack of Specific Medical Guidance: The advice is very generic and does not account for the nuanced and personalized nature of medical advice required for patients with Parkinson's disease. For example, patients might have specific medication regimens or conditions that could affect their ability to tolerate heat or sun exposure. | | | | | |
|  | GPT 4 | Não sou médico, mas posso oferecer algumas informações gerais sobre a doença de Parkinson e a Estimulação Cerebral Profunda (ECP). No entanto, é essencial que você consulte o seu médico ou profissional de saúde para aconselhamento personalizado sobre as suas atividades diárias.  Em geral, a doença de Parkinson não impede que você vá à praia ou tome sol. No entanto, após a cirurgia de Estimulação Cerebral Profunda, é importante tomar precauções adicionais.  Cicatrização: Certifique-se de que a incisão cirúrgica esteja completamente curada antes de se expor ao sol ou entrar na água. Isso é importante para evitar infecções e promover uma cicatrização adequada.  Exposição ao sol: Use protetor solar e roupas adequadas para proteger a pele, especialmente nas áreas onde você teve a cirurgia. A exposição ao sol pode causar irritação na pele e prejudicar a cicatrização.  Natação e atividades aquáticas: Após a ECP, pergunte ao seu médico quando você pode começar a nadar ou participar de outras atividades aquáticas. Ele ou ela provavelmente recomendará que você espere até que a incisão esteja totalmente curada.  Cuidado com o dispositivo: Se você tiver um dispositivo implantável, como um neuroestimulador, é importante tomar precauções para protegê-lo. Consulte o seu médico ou o fabricante do dispositivo para saber se existem recomendações específicas sobre exposição à água ou a altas temperaturas.  Lembre-se de que a doença de Parkinson pode afetar a sua mobilidade e equilíbrio. Portanto, tenha cuidado ao se deslocar pela praia ou entrar na água. Peça a ajuda de amigos ou familiares, se necessário, e siga as recomendações do seu médico. | 1 | 1 | - |
| 79 | (EN) I was submitted to a DBS surgery for Parkinson’s disease.  **My wound is not closing. What should I do?** | | | |  |
|  | GPT 3.5 | If you are experiencing issues with your surgical wound not closing after a deep brain stimulation (DBS) surgery for Parkinson's disease, it is important to seek medical attention as soon as possible. Here are a few steps you can take:  Contact your surgeon or neurologist: Your healthcare provider is the best person to advise you on what to do in this situation. Reach out to them immediately and explain your concerns.  Keep the wound clean and dry: Until you can see a healthcare provider, it's important to keep the area around your wound clean and dry. Wash your hands before touching the wound, and avoid getting it wet.  Avoid putting pressure on the wound: Try not to put pressure on the wound or do any activities that might cause it to open further.  Watch for signs of infection: Keep an eye out for any signs of infection, such as redness, swelling, warmth, or discharge from the wound. If you notice any of these symptoms, seek medical attention immediately.  Follow your healthcare provider's instructions: Your healthcare provider will give you instructions on how to care for your wound and what steps to take if it does not close. Be sure to follow their instructions carefully.  In summary, if you are experiencing issues with your surgical wound not closing after a DBS surgery for Parkinson's disease, it's important to seek medical attention as soon as possible. Contact your healthcare provider and follow their instructions carefully to ensure proper healing. | 1 | 2 | 2 |
|  | GPT 4 | I'm not a doctor, but I can try to help you with some general advice. If your wound isn't healing properly after Deep Brain Stimulation (DBS) surgery, it's important to seek medical attention as soon as possible. Wound healing complications can lead to infection or other serious is Here are some general steps to consider, but remember to consult with a healthcare professional:  Keep the wound clean: Make sure to clean the wound gently with mild soap and water daily, as directed by your healthcare provider.  Protect the wound: Keep the wound covered with a clean, dry bandage as recommended by your healthcare provider. Avoid picking or scratching the wound.  Elevate the area: If possible, try to elevate the affected area to minimize swelling and promote healing.  Watch for signs of infection: Monitor your wound for any signs of infection, such as increased redness, swelling, pus, or discharge, as well as fever or chills. If you notice any of these symptoms, contact your healthcare provider immediately.  Follow your healthcare provider's instructions: It's crucial to follow any post-operative care instructions provided by your healthcare provider, including taking prescribed medications, attending follow-up appointments, and following any activity restrictions.  Again, it's important to consult with a healthcare professional about your specific situation, as they can provide personalized advice and guidance for your recovery. | 1 | 2 | 2 |
| 80 | (PT) Tenho doença de Parkinson e fui submetido a Estimulação Cerebral Profunda.  **A minha ferida não está a fechar. O que devo fazer?** | | | |  |
|  | GPT 3.5 | Se a ferida não está a fechar, é importante procurar atendimento médico imediatamente. Você pode entrar em contato com o seu médico responsável pelo seu tratamento ou ir a um serviço de emergência hospitalar para avaliação. O processo de cicatrização pode ser afetado pela doença de Parkinson, pelo uso de medicamentos ou pela própria cirurgia de Estimulação Cerebral Profunda. Por isso, é importante que você seja avaliado por um profissional de saúde para determinar a causa do problema e o tratamento adequado. Alguns cuidados podem ser tomados para ajudar no processo de cicatrização, como manter a ferida limpa e seca, evitar movimentos bruscos que possam tensionar os pontos, e seguir as instruções de repouso e cuidados pós-operatórios indicados pelo seu médico.  No entanto, somente um profissional de saúde capacitado poderá avaliar a situação e orientar o tratamento adequado para o seu caso específico. Portanto, não hesite em buscar ajuda médica o mais rápido possível. | 2 | 2 | - |
|  | GPT 4 | Não sou médico, mas posso tentar oferecer algumas sugestões gerais. Se a sua ferida não está a fechar após a cirurgia de Estimulação Cerebral Profunda (ECP), é importante que consulte o seu médico ou neurocirurgião o mais rápido possível. Eles poderão avaliar a situação e determinar a melhor forma de tratar a ferida. Entretanto, aqui estão algumas dicas gerais que podem ajudar: Mantenha a área limpa e seca: Lave a ferida com água morna e sabão neutro, e seque-a cuidadosamente com uma toalha limpa. Evite esfregar a área.  Evite tocar na ferida com as mãos sujas: Lave sempre as mãos antes de tocar na área afetada para reduzir o risco de infeção.  Troque os curativos conforme indicado pelo seu médico: Se o seu médico lhe recomendou o uso de curativos, certifique-se de trocá-los conforme as instruções.  Não puxe crostas nem fios de sutura: Isso pode atrasar a cicatrização e aumentar o risco de infeção.  Evite atividades que possam irritar a área: Reduza atividades que causem pressão, atrito ou estresse na área da ferida.  Informe o seu médico se notar sinais de infeção: Se a área apresentar sinais de infeção, como vermelhidão, inchaço, dor intensa ou secreção de pus, contacte o seu médico imediatamente.  Lembre-se de que esta informação não substitui o aconselhamento médico profissional. É fundamental que fale com o seu médico para obter orientações específicas sobre o seu caso. | 1 | 2 | 1 |
